# Impact of transport source (dairy farms vs. collection center) and post-arrival housing in combination with prophylactic antibiotic treatment on performance, lung health and microbiota of veal calves

**DOI:** 10.3389/fvets.2026.1715667

**Published:** 2026-03-02

**Authors:** F. Marcato, D. Schokker, N. Stockhofe-Zurwieden, G. Gort, C. A. Jansen, M. Wolthuis-Fillerup, J. Osaro John, A. C. Strappini, M. S. Gilbert, W. J. J. Gerrits, C. G. Van Reenen

**Affiliations:** 1 Wageningen Livestock Research, Animal Health and Welfare Group, Wageningen, Netherlands; 2 Wageningen Bioveterinary Research, Lelystad, Netherlands; 3 Biometris, Wageningen University & Research, Wageningen, Netherlands; 4 Cell Biology and Immunology Group, Wageningen University & Research, Wageningen, Netherlands; 5 Animal Nutrition Group, Wageningen University & Research, Wageningen, Netherlands

**Keywords:** antibiotics, health, immunity, microbiota, source, transport

## Abstract

Transport via a collection center and early life administration of antibiotics are two major challenges faced by calves in current veal husbandry management. The main aim of this proof of concept study was to investigate the effects of these two factors on fecal microbiota, lung immunity, and overall health of veal calves. The study had a 2 × 2 factorial design with the following factors: source of calves [transport via a collection center (CC) or direct transport from dairy farms (DF)], and early-life administration of antibiotics (AB or no AB). The hypothesis was that direct transport and AB use may positively affect the health of calves. A total of 89 bull calves and 5 heifers, aged two to 4 weeks, and with average 46.4 ± 5.1 kg body weight (BW) at arrival were included in the study. The study was performed in two consecutive batches, and calves were followed for a period of 8 weeks. At arrival, animals were housed in groups of three calves/pen divided over six rooms. Calves from the DF were housed in separate rooms, whereas CC calves were housed in multiple pens per room. The AB-treatments received an oral antibiotics/antiphlogistic treatment via the milk replacer on day 4 after arrival. On day 21 and 45, fecal samples and broncho-alveolar lavage fluid (BALF) were collected, whereas nasal swabs were collected on day 7 for microbiota analysis. Blood samples were collected on day 1, 7, 21, 35, 45, and 51 for the complete hematological profile and immune cells. Body weights were recorded upon arrival, and day 28 and 49, and clinical observations were conducted twice a week throughout the experiment. Post-mortem examinations were also performed. Calves sourced from CC and not receiving AB (CC_No AB) had the lowest percentage of alveolar macrophages, the highest incidence of clinical problems and the lowest BW at the end of the trial. Fecal Shannon index and Pielou’s evenness was reduced in CC_AB calves compared to all other treatments. Calves sourced via CC showed a higher lung/heart ratio and more abnormalities in the lungs compared to DF calves. Overall, this study showed that transportation via a CC without subsequent treatment with AB represented the greatest challenge on clinical health, immunity, and fecal microbiota of veal calves.

## 1 Introduction

Bovine respiratory disease (BRD) complex is one of the most important diseases affecting veal calves, leading to clinical health problems and economic losses due to hampered growth (1). It is also a primary reason for a high antibiotic use in veal production (2, 3). In the veal sector, calves from different dairy farms are typically transported around 2 weeks of age to collection centers, and from there to specialized veal farms (4). This practice results in a high infection pressure and, correspondingly, a high incidence of BRD (5). Bovine respiratory disease is a multifactorial disease involving multiple pathogens, including *Mycoplasma* spp., *Pasteurella multocida*, *Mannheimia* spp., bovine viral diarrhea virus and bovine respiratory syncytial virus (6). Since bacterial pathogens play a significant role in BRD in veal calves, antibiotics are often administered, both as group treatments (most frequently within the first 2 months upon arrival at the veal farm), and in individual calves. However, a high antibiotic use can lead to the development of antimicrobial resistance, which is a global threat (7, 8). Therefore, it is important to investigate factors that might help prevent or reduce respiratory problems, and consequently, antibiotic use, in veal calves.

Gut microbiota are of great importance for the health of the host. They support the fermentation in the rumen or large intestine (9), prevent the growth of harmful microorganisms (10), and maintain the barrier between intestinal contents and the body (11). Moreover, the extensive communication between components of the gut microbiota and the rest of the body can affect the host’s immune system in terms of development and functionality, both local as well as systemic (12, 13). Disturbances of the gut microbiota (dysbiosis) can impair immune function and increase the risk of diseases, such as BRD. In contrast, beneficial changes in composition of the microbiome (e.g., higher number of beneficial bacteria) can have a positive effect on disease resistance and health (14). Knowledge of factors that affect the gut microbiome, especially those influencing the immune system and health, can form the basis for novel strategies to improve the health of farm animals, including veal calves. Recent research in humans and laboratory animals indicates that the gut microbiota particularly affects lung immunity and may play a role in resistance against respiratory infections (15, 16). This concept is referred to as gut-lung axis that allows bidirectional communication between the gut and the lungs (17). As seen in Gilbert et al. (18), the microbiota of veal calves may also be functionally linked to lung microbiota and lung immunity, indicating the existence of a similar gut-lung axis.

At present the physiological pathways involved in the interaction between gut microbiome, immunity and general health are not well understood in veal calves. Moreover, there is a knowledge gap on how the putative bidirectional communication between lungs and gut might be affected by prevailing challenges that calves are exposed to during the production cycle (e.g., sourcing, administration of group treatment with antibiotics). Knowledge regarding the effects of calf source and antibiotic treatment in early life can ultimately provide the basis for developing strategies and interventions that increase the resilience of calves to respiratory infections, possibly by changing the gut microbiota of calves. These factors were therefore investigated in the current study. We hypothesized that direct transport of calves from a dairy farm to an experimental farm rather than via a collection center, as well as a group antibiotic treatment upon arrival at the experimental farm, would positively affect calf health. Moreover, the manipulation of the gut microbiome through these interventions would allow us to evaluate associations between the (gut and fecal) microbiota and measurements of lung immunity, to increase our understanding of the gut-lung axis in veal calves. In this experiment, treatment levels were chosen to represent, for the first time, the extremes of two methods for sourcing calves, and two antimicrobial treatment strategies, respectively. This allowed us to prove the concept that our interventions of interest are relevant for calf health, and would merit further study under practical conditions. The overall aim of the current proof of concept study was as to investigate the effects of different methods of sourcing veal calves and different antimicrobial treatment strategies on calf health, gut microbiota, immune cell phenotype and functionality in the blood and lung fluid.

## 2 Materials and methods

The current study was designed as a proof of concept study under controlled experimental conditions at the experimental facility CARUS, at Wageningen University and Research. The experiment was approved by the Central Committee on Animal Experiments (The Hague, The Netherlands; approval number AVD1040020185828) and the local Animal Welfare Body (approval number 2017. W-0017).

### 2.1 Experimental design

To investigate the effects of mixing of calves from different sources and the use of antibiotics on the gut-lung axis the experiment was designed as a complete 2 × 2 factorial design, resulting in the following 4 treatment combinations:

1) DF_No AB: calves transported directly from a dairy farm to the experimental farm and not receiving a prophylactic group antibiotic treatment in the first week after arrival.
2) DF_AB: calves transported directly from a dairy farm to the experimental farm, receiving a prophylactic group antibiotic treatment in the first week after arrival.
3) CC_No AB: calves transported first from a dairy farm to a collection center (where they were mixed with other calves), then from the collection center to the experimental farm; not receiving a prophylactic group antibiotic treatment in the first week after arrival.
4) CC_AB: calves transported first from a dairy farm to a collection center (where they were mixed with other calves), then from the collection center to the experimental farm; receiving a prophylactic group antibiotic treatment in the first week after arrival.

*Group antibiotic treatment*: the calves assigned to the DF_AB and CC_AB treatment groups received antibiotic with the milk replacer (MR) [oxytetracycline (Dopharma Veterinaire Farmaca B.V.; the Netherlands); 1 g/calf/feeding] in combination with aspirin (1 g/calf/feeding) on day 4 after arrival. This was administered over eight feedings for 4 days.

The experiment lasted 8 weeks, and was performed in two batches. The first 8 weeks post-transport represent the most vulnerable period for calves, especially with regard to gastrointestinal and lung problems, and this was the justification for the duration of the experiment.

### 2.2 Animal and housing

**Figure 1:**
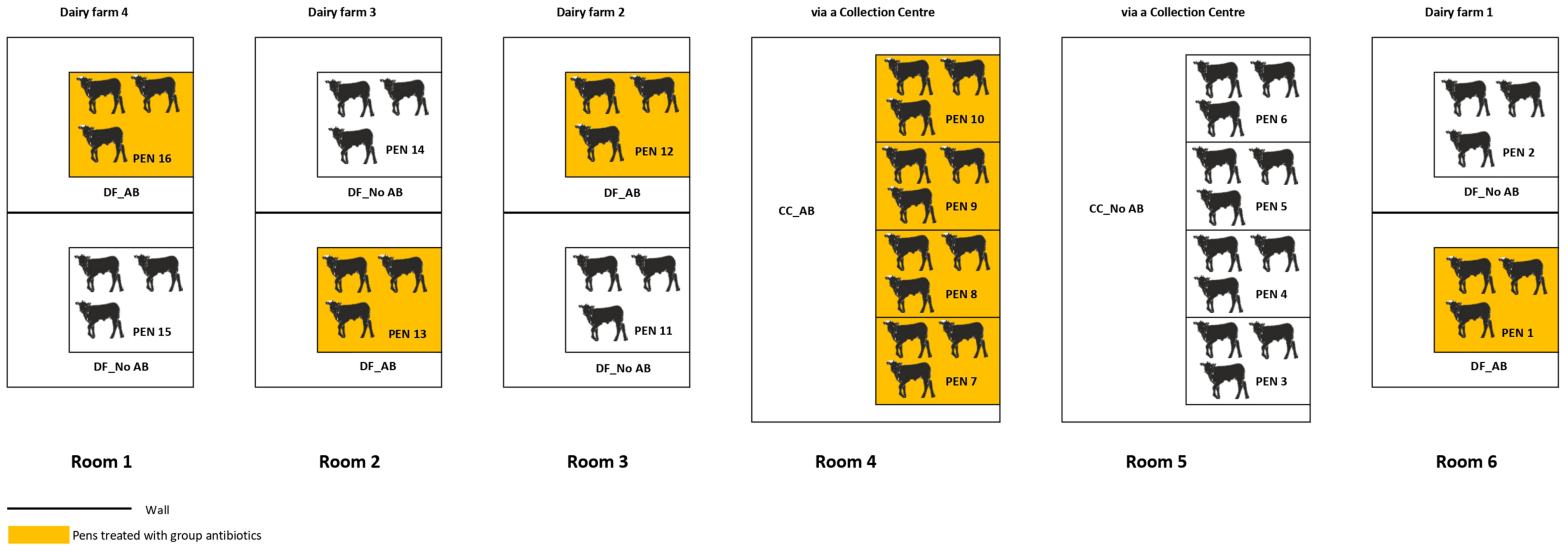
Distribution of calves in each treatment at the experimental farm (DF_AB = calves originating from dairy farms receiving group antibiotics after arrival; DF_No AB = calves originating from dairy farms not receiving group antibiotics after arrival; CC_AB = calves originating from a collection center receiving group antibiotics after arrival; CC_No AB = calves originating from a collection center not receiving group antibiotics after arrival). Four rooms housed calves sourced directly from dairy farms whereas two rooms with fours pens housed calves sourced from a collection center. Upon arrival calves originating from the same dairy farm were housed together in the same room. Pens colored in orange received group treatment with antibiotics on day 4 post-arrival. The bold line indicates the presence of a wall separating the room into two separate environments.

A total of 89 unweaned bull calves (four crossbreds and 85 Holstein-Friesian) and five heifers (one Red Holstein-Friesian and four Holstein-Friesian) with an average body weight (BW) of 46.4 ± 5.1 (mean ± SD) kg and average age of 16 ± 2.7 days at arrival, were included in the study. The age difference between the youngest and oldest calf was 11 days. However, the treatment groups were all balanced in terms of age (average age was always 16 days). This age range was in line with the current commercial practices in the Netherlands (19). The number of calves included in our study was a compromise between, on the one hand, available resources and practical feasibility of the experiment and, on the other, obtaining a representative sample with sufficient statistical power. Calves were transported in two batches from four Dutch dairy farms or from one Dutch collection center to the experimental facility. The first batch (*n* = 48 calves) arrived at the experimental facility on October 31st 2022, whereas the second batch (*n* = 46 calves) arrived on January 16th 2023. The background and management of calves on the source farms and at the collection center was unknown to the researchers. Per batch, calves were housed in 16 pens, which were divided over six rooms (see Figure 1). Calves transported from a collection center were housed in two rooms, each containing each four adjacent pens belonging to the same AB treatment. Calves directly transported from the dairy farms were housed in four rooms each containing two pens (one per treatment group). These DF rooms were physically divided into two separate environments by a wall to minimize the spreading of pathogens between pens belonging to different antibiotic treatment groups. In addition, boots and coveralls were changed when moving to another pen within a DF room. Calves were housed in groups of three per pen (Figure 1). The pens (2.40 × 2.43 m) had a concrete slatted floor with a rubber top layer and a brush and a calf nipple dummy were provided for enrichment. The lights were on for 12 continuous hours a day.

### 2.3 Diet and feeding

Upon arrival at the experimental facility, calves received around 1 L of electrolytes. From day 2 onwards, calves were fed twice a day at 07:00 and 16:00 h. Calves were fed commercial milk replacer (Denkaveal starter in the first 2 weeks and after Gold milk replacer of Denkavit B.V., Voorthuizen, the Netherlands), concentrates (provided as flakes; Denkaveal Mueslibar Extra of Denkavit B.V., Voorthuizen, the Netherlands) and chopped straw according to a practical feeding schedule of Denkavit B.V. (see Supplementary Tables S1, S2). Calves were fed equal amounts of milk replacer, which was provided individually at a drinking temperature of 42.5–43 °C. Thereafter, solid feed (a concentrate to straw ratio of 10:1 on product basis) was provided per pen in a stainless-steel trough, and refusals were quantified. The feed leftover at pen level were recorded by weighing the residual pelleted feed and chopped straw weekly. Potential milk leftovers per calf were recorded after every feeding. Water was provided *ad libitum* through an automatic drinking bowl during the whole study.

### 2.4 Measurements

A total of 80 calves (40 animals/batch) were randomly sampled for blood, rectal swabs and broncho-alveolar lavage fluid (BALF) collection during the experiment. The same calves were sampled at each sampling moment. These animals were also selected for post-mortem measurements. Clinical health measurements were performed on all 89 calves.

#### 2.4.1 Clinical health

Individual clinical health assessments of all calves were performed by the same two trained researchers twice a week. Rectal temperature (RT) was taken and clinical signs were scored in accordance with the protocol published by Marcato et al. (19) (Supplementary Table S3).

#### 2.4.2 Performance

Calves were individually weighed at arrival (day 0), halfway through the study (day 28) and at the end of the study (day 49). The average solid feed intake of calves per pen was determined by weighing the feed provided and the leftover feed every week. At the end, the average daily feed intake (ADFI) per pen, average daily gain (ADG) per calf and the feed conversion ratio (FCR) per pen, were calculated. The use of medications (including antibiotics and other medical treatments) was recorded for each calf throughout the experiment. Individual treatments were administered only when the veterinarian deemed it appropriate after clinical inspection of the animals.

#### 2.4.3 Hematology and immune cells in the blood

Blood samples were taken from the jugular vein of calves on day 1, 7, 21, 35, 45, and 51 of the experiment. A total of three Vacutainer tubes (Vacuette, Grenier BioOne) per calf were taken: 10 mL EDTA and 5 mL serum for hematological profile analysis, and 5 mL EDTA for whole blood flow cytometry analysis (FACS; up to day 45 only). The SMART V5 laser hematology analyzer (A. Menarini Diagnostics) was used for haematological analyses, including white blood cell (WBC) count, red blood cell count, hemoglobin (Hb), hematocrit (Hct) and mean corpuscular volume (MCV), as well as platelet count. Samples collected on day of autopsy were taken before the calves were euthanized.

Quantification of numbers of immune cells in whole blood was performed using BD Trucount™ Tubes (BD Biosciences, San Jose, USA) according to manufacturer’s instructions. Briefly, 200 μL EDTA blood was mixed with 40 μL Transfix® (Invitrogen) and diluted 1:50 in FACS buffer (PBS containing 0.5% bovine serum albumin and 0.1% sodium azide). Antibody mix (20 μL) was added to Trucount tubes followed by 50 μL diluted blood and incubated for 15 min at room temperature in the dark. The antibody panel included mouse anti-bovine CD45-AF647, mouse anti-bovine CD8α-PB, mouse anti-bovine CD4-AF700, mouse anti-bovine CD335-PE, and mouse anti-bovine WC1-FITC. All antibodies were obtained from Biorad (Veenendaal, the Netherlands). Erythrocytes were lysed using 450 μL lysis buffer (BD Biosciences, Drachten, the Netherlands) before acquisition on a CytoFLEX LX (Beckman Coulter, Indianapolis, USA). Data were analyzed with FlowJo v10.10.0 (Tree star Inc., Ashland, OR, USA), and absolute cell counts were calculated.

#### 2.4.4 Immune parameters in BALF

On days 21 and 45 of the experiment BALF samples were collected via the ventral nasal meatus, following the protocol described by Antonis et al. (3). A total of 10–20 mL of BALF was obtained from each calf after introduction of 30 mL PBS using a sterile silicon tube with an diameter of either 3 mm or 6 mm, and an external diameter either 7 mm or 10 mm, depending on the age of the calves. Immediately after collection, the samples were stored on ice and then divided into two subsamples that were used to analyze alveolar macrophages or for the functional assays. Samples were filtered through a 100 μm cell strainer (Corning), counted and for each staining approximately 5 × 10^5^ cells alveolar macrophages were used. Cells were transferred to a round bottom 96- well plate (NUNC) and were washed once with 200 μL of FACS buffer. Next, 30 μL antibody mix containing the mouse-anti-bovine MHC-II-PE and unconjugated mouse-anti-bovine-CD11c was added to each well. After 20 min on ice, the cells were washed with either 30 μL of rabbit-anti-mouse-IgM-BV421 (cd11c stained cells) or FACS buffer (MHC-II stained cells). After another 20 min on ice the cells were washed, resuspended in 150 μL FACS buffer and flow cytometry was performed. Samples were measured using a CYTOFLEX LX (Beckman Coulter) and approximately 100,000 cells were recorded per sample. Macrophages identification was performed with FlowJo 10.10.0 software (Tree Star Inc., Ashland, OR, USA).

The second BALF sample was used for functional assays evaluating reactive oxygen species (ROS) production and phagocytosis. Samples were centrifuged for 10 min at 1500 rpm at 4 °C, treated with acetate kinase (ACK) buffer to lyse red blood cells, and centrifuged again. Cells were resuspended in PBS, counted, and adjusted to 2 × 10^6^ viable cells/ml in DPBS (Gibco). Subsequently, 2 × 10^5^ cells in 100 μL were seeded per well in flat-bottom 96-well plates (Greiner, art.nr. 655,090 and incubated overnight at 37 °C and 5% CO₂).

For phagocytosis assays, cells were incubated with 1 × 10^7^ fluorescein-labeled *Escherichia coli* (K-12 strain) Bioparticles™ (Thermo Fisher, catalogue number E2861) for 60 min at 37 °C. Phagocytosis was stopped using 4% paraformaldehyde, followed by PBS washes, and fluorescence was measured using a Clariostar plate reader (ISOGEN Life Science, Utrecht, the Netherlands) according to the manufacturer instructions.

ROS production was assessed using the 2′,7′-dichlorofluorescin diacetate (DCFH-DA, Sigma, D6883) assay. Briefly, cells were adjusted to 1 × 10^6^ viable cells/ml in RPMI, seeded at 5 × 10^4^ cells per well, incubated for 1 h at 37 °C/5% CO_2_, and stimulated with 50 μL PBS or 50 μL PMA (Phorbol-Myristate-Acetate) prior to DCFH-DA incubation. Fluorescence was measured after 60 min in a Clariostar plate reader according to the manufacturer instructions.

#### 2.4.5 Microbiota

On day 7, nasal swabs (Minitip Flocked swab with 80 mm breakpoint in dry tube, Copan Diagnostics, Brescia, Italy) were collected to analyze the microbiota of the nasal mucosa.

Rectal swabs (Pharma Regular Applicator Flocked-tipped in Dry Tubes, Copan Diagnostics, Brescia, Italy) were collected on days 21 and 45 post-arrival of calves at the experimental facility. Samples were stored at −80 °C pending fecal microbiota analysis. The 16S rRNA gene was targeted, in the V5 and V6 region of the rectal swabs, using the following primers 5′-ATTAGATACCCTGGTAGTCC-3′ and 5′-TCACRRCACGAGCTGACGACA-3′. In addition, the V1 and V3 regions were targeted to gain more insight into archaea, where the following primer pair was used ‘5-TCCGGTTGATCCYGCBRG-3’ and 5’-GCTACGRRYGYTTTARRC-3′. The 16S hyper-variable region V5–V6 was amplified by 25 cycles of three parallel PCR runs, which were then pooled to maximize the diversity detected. In a separate set of PCR reactions, ITS1 was targeted to detect fungi, by using the primers 5′-CTTGGTCATTTAGAGGAAGTAA-3′ and 5′-GCTGCGTTCTTCATCGATGC-3′. PCR products were checked on a 2200 Tape station and all amplicons were subsequently equimolar pooled, and a barcode was added to each sample prior to sequencing. Sequencing was performed using a version 3 paired-end 300- base-pair sequencing kit on a MiSeq sequencer (Illumina Inc., San Diego, California, USA). Negative controls were included in DNA extractions and PCRs to identify potential (cross) contamination between samples during processing and to confirm the sterility of reagents. A mock community (ZymoBIOMICS™ Microbial Community Standard containing bacterial and yeast community) was also included in the sequencing run, as a positive control to confirm specificity.

In addition to fecal swabs, microbiota were analyzed in jejunal digesta samples from batch 2 calves. Jejunal digesta were collected in 2 mL tubes during the autopsy carried out on days 50 and 51, and samples were stored at −80 °C prior to the analyses.

The microbiota in the nasal swabs and jejunal digesta were typed by amplifying the full-length 16S rRNA gene using Oxford Nanopore technology. The primers 27F and 1492R (5’-AGRGTTTGATYHTGGCTCAG-3′ and 5’-TACCTTGTTAYGACTT-3′) were used for this amplification, each modified with a Nanopore-compatible sequence tag to facilitate compatibility with the Nanopore PCR barcoding system. All PCR reactions were performed in triplicate to mitigate potential amplification bias.

To prepare the sequencing libraries, a two-step PCR approach was utilized, beginning with a 25-cycle gene-specific amplification using Q5® High-Fidelity 2X Master Mix to ensure high sequence accuracy. The resulting amplicons were purified using Agencourt® AMPure® XP beads at a 0.8 ratio and subsequently barcoded via a secondary 10-cycle PCR to allow for sample multiplexing. A final bead-based purification was performed to yield high-purity, indexed libraries ready for downstream sequencing applications [the full detailed description is written in Marcato et al. (20)]. The individually barcoded amplicons were then normalized based on quantification to ensure an equal amount of each sample was loaded onto an Oxford Nanopore Technologies PromethION flow cell. This normalization step is crucial for generating balanced sequencing data across samples. The raw sequencing data were base-called using the Dorado software, followed by demultiplexing to separate reads according to their barcodes. An additional round of demultiplexing using Porechop was performed to ensure optimal accuracy, resulting in a FastQ file ready for downstream analysis. The sequence data were analyzed using Coatofarms,^1^ producing relative abundance tables. These tables were then used to generate a *phyloseq* object for further analysis of the microbial community.

#### 2.4.6 Post-mortem measurements

Post-mortem examination was carried out over 2 days (days 50 and 51). After jugular blood sampling, calves were euthanized by injecting pentobarbital (Euthasol 40% ^®^, Dechra, the Netherlands) via the jugular vein followed by exsanguination via the carotid artery and jugular vein. Gross pathological findings were systematically recorded and weights of lungs and hearts were taken. The respiratory tract, including the lungs, was examined and scored for abnormalities in trachea, bronchi, lung lymph nodes and lung tissue (score 0 = normal; score 1 = presence of abnormalities). The extent and type of tissue changes were recorded. Jejunum content samples were collected into Eppendorf tubes, immediately freeze-dried in liquid nitrogen and then stored at −80 °C for further analyses.

### 2.5 Statistical analyses

Statistical analyses were performed using SAS version 9.4 (SAS Institute Inc.), with the exclusion of part of the microbiota data, which were performed in R. Residuals were always checked for normality and homogeneity of variance, and variables were log-transformed when needed. Normality was assessed via histogram, Q-Q plot and skewness and kurtosis values. Homogeneity of variances was assessed via residuals vs. fitted plots. Blood, BALF, and Alpha diversity data (Richness, Shannon index, and Pielou’s evenness) of fecal microbiota were analyzed across timepoints with a linear mixed model (LMM, employing PROC MIXED) for continuous data or generalized linear mixed model (GLMM, employing PROC GLIMMIX) with the beta distribution for continuous proportional responses. The model (model 1) included fixed effects of batch (1 or 2), source (CC or DF), AB (yes or no), day (day 21 and 45 for BALF data; day 7, 21, 35, 45, 51 for blood data), and the interactions Source × AB, Source × Day, AB × Day, and Batch × Day. The model also included, depending on the response variable, random effects for batch × room, batch × room × pen, and batch × room × pen × animal. Moreover, the variance components were allowed to be different for calves originating via the CC and the ones directly transported from DF. Interactions that were not significant (*p* > 0.05) were removed with the backward procedure. In the model used for blood data, a first-order autoregressive model (based on the actual distance between time points) was adopted to introduce correlation in the model between repeated measurements on the same animal. Approximate *F*-tests (21) were used for fixed effects. Subsequent pairwise comparisons were done with Fisher’s least significant difference method.

A part of the analysis of sequencing data on fecal microbiota was performed using R version (v4.3.2), RStudio (v2023.12.0 Build 369), and functions of R packages *phyloseq* (v1.46.0) and *mia* (v1.13.34). Beta diversity data were rarefied using the *rarefy_even_depth* function. For the linear models, the *lmer* function was used, and the model included Source, AB, batch, day, Source × AB, Source × day, AB × day, and batch × day as factors and pen as random factor.

A part of blood immune variables, microbiota in jejunum and nasal swabs were only measured for batch 2. These variables were analyzed with model 1, with the exclusion of batch effects.

Clinical health scores recorded at arrival (timepoint 0) were analyzed as binary variables (0 = normal and 1 = presence of a health problem) with a generalized linear mixed model (GLMM) comprising a logit link function and Bernoulli distribution. Rectal temperature at arrival was also expressed as a binary variable (0 = calves with temperature <39.5 °C; 1 = calves with fever [temperature ≥39.5 °C); Garcia et al. (22)]. The model (model 2) included fixed effects for batch (1 or 2), source (CC or DF), and the interaction Source × Batch. The random component was the same as in the previous analyses.

Body weight (BW), which was measured upon arrival, was analyzed using a LMM including the same factors mentioned in model 2, whereas BW, measured on days 28 and 49, and ADG were analyzed using a LMM including the following fixed effects (model 3): batch (1 or 2), source (CC or DF), AB (yes or no), and the interaction Source × AB. The model also included BW on day 0 as a covariate. The random component was the same as the previous analyses. Due to the low incidence of clinical health problems per observation day after day 0 we calculated an overall prevalence of clinical health problems across all time points. This new variable was calculated at calf level, and it was analyzed with model 3.

Individual use of antibiotics and other medical treatments during the experiment was expressed as a binary variable (0 = calf never treated individually with antibiotics or other medicines; 1 = calf treated at least once with antibiotics or other medicines) and thus analyzed using the GLIMMIX procedure with binary distribution and model 3.

Heart and lung weights obtained during the autopsy were expressed as continuous variables and analyzed with LMM model 3. Presence (score 1) or absence (score 0) of abnormalities in lung lobes 1 and 2, and presence (score 1) or absence (score 0) of enlarged tracheal lymph nodes, pus in the trachea, pus and mucus in bronchi, were analyzed with the GLIMMIX procedure with a binary distribution and model 3. A total lung score was also calculated from the individual lobe scores (on a scale from 0 = no abnormal lobes to 10 = abnormalities in all lobes). Total lung score was analyzed with LMM and model 3.

In all analyses, effects with *p* ≤ 0.05 were considered significant. Data are reported as raw means.

## 3 Results

### 3.1 Clinical health

There was no difference in clinical health upon arrival between calves transported via CC and those originating directly from the DF (Supplementary Table S5). However, the percentage of calves showing loose or liquid manure (*p* = 0.05), eye discharge (*p* = 0.02), and drooped ears (*p* = 0.02) at arrival was higher in batch 1 than in batch 2. The percentage of calves with sunken eyes was higher in batch 2 compared to batch 1 (*p* < 0.01; Supplementary Table S5).

Supplementary Figures S1A–F show the prevalence of clinically ill calves per treatment over the entire experiment. The prevalence of calves with fever and loose or liquid manure was higher for the CC_No AB calves than for calves of the other treatments (*Δ* = 9.2% for fever and Δ = 13.1% for loose or liquid manure on average; Source × AB interaction *p* ≤ 0.05; Table 1). Calves originating from the CC had a higher percentage of coughing (Δ = 11.8%; *p* < 0.01) and nasal discharge (Δ = 14.4%; *p* < 0.01) compared to calves directly transported from DF (Table 1). The CC treatment tended to have a higher percentage of sunken eyes (Δ = 10.3%; *p* = 0.10) compared to the DF treatment (Table 1).

**Table 1:** Effects of source (direct transport from the dairy farms (DF) and subsequent separate housing per dairy farm or transport via a collection center (CC) and subsequent mixed housing) and the prophylactic application of an antibiotic treatment in early-life (AB; yes or no) on cumulative percentage of calves with clinical problems during the 8-week experiment (values are reported as raw means and their pooled SEM^1^).

Parameter^1^	Dairy farm (DF)		Collection center (CC)			*p*-value			
	No AB	AB	No AB	AB	SEM	Source	AB	Batch	Source×AB
No. of calves	23	23	24	24					
Fever	2.9^b^	2.9^b^	13.3^a^	6.4^b^	1.2	<0.01	0.02	0.37	0.04
Navel inflammation	13.0	23.2	26.7	19.4	5.5	0.48	0.75	0.70	0.16
Joint inflammation	0.3	6.7	1.1	0.3	1.3	0.22	0.18	0.45	0.11
Loose or liquid manure	8.1^b^	9.6^b^	21.9^a^	8.6^b^	2.2	0.06	0.18	0.06	0.05
Coughing	1.2	1.2	16.1	10.0	1.6	<0.01	0.13	0.25	0.13
Eye discharge	19.7	20.6	32.5	21.4	4.2	0.16	0.26	<0.01	0.19
Sunken eyes	44.3	42.6	55.5	51.9	2.71	0.10	0.47	<0.01	0.84
Drooped ears	14.1	15.1	17.6	15.8	2.95	0.51	0.92	<0.01	0.68
Nasal discharge	7.6	4.9	20.7	20.6	2.88	<0.01	0.77	<0.01	0.65

1 SEM, standard error of the mean.^a,b^LSM within a factor and row lacking a common superscript differ (*P* ≤ 0.05).

### 3.2 Performance

Calves from batch 1 had a higher BW at arrival compared to calves of batch 2 (47.6 vs. 45.3 kg; *p* = 0.02; Table 2). Furthermore, calves directly transported from DF were heavier than calves originating from the CC (Δ = 3 kg; *p* = 0.02; Table 2). Compared to the other treatment groups, CC_No AB calves had a reduced ADG between day 0 and day 28 (−91 g/d on average; *P* interaction = 0.05) and tended to have a reduced ADG between day 0 and day 49 (*P* interaction = 0.07). At day 49, BW of CC_No AB calves was 4.4 kg lower compared to the other treatment groups (Source × AB interaction; *p* < 0.01; Table 2). Prophylactic administration of AB increased ADG from day 0 to 28 (661.5 g/d vs. 619 g/d; *p* = 0.05) and from day 0 to day 49 (784 g/d vs. 735 g/d; *p* = 0.03; Table 2).

**Table 2:** Effects of source (direct transport from the dairy farms (DF) and subsequent separate housing per dairy farm or transport via a collection center (CC) and subsequent mixed housing) and the prophylactic application of an antibiotic treatment in early-life (AB; yes or no) on body weights and average daily gain (ADG) of calves measured at three timepoints (values are reported as raw means and their pooled SEM^1^).

Parameter	Dairy farm (DF)	Collection center (CC)	SEM^1^	*p*-value			
				Source	AB	Batch	Source × AB
BW (kg), day 0	48.0	45.0	0.6	0.02	–	0.02	–

Parameter	Dairy farm (DF)		Collection center (CC)		SEM	*p*-value			
	No AB	AB	No AB	AB		Source	AB	Batch	Source × AB
BW (kg)									
Day 28	66.9	66.9	61.2	63.9	1.1	0.16	0.06	0.29	0.10
Day 49	85.1^b^	84.1^b^	80.2^a^	84.6^b^	1.0	0.03	0.08	0.34	<0.01
ADG (g/d)									
Day 0–28	665^b^	666^b^	573^a^	657^b^	29.7	0.21	0.05	0.66	0.05
Day 28–49	889	907	820	914	40.8	0.54	0.16	0.18	0.33
Day 0–49	779	786	691	781	25.8	0.13	0.03	0.78	0.07

1 SEM, standard error of the mean. ^a,b^LSM within a factor and row lacking a common superscript differ (*P* ≤ 0.05).

Individual antibiotic and medical treatment during the experiment was not affected by treatment. Seven CC_AB calves and 2 DF_AB calves were treated individually with antibiotics at least once. Seven CC_AB calves, 3 CC_No AB calves, and 1 DF_AB calf were treated with medicines other than antibiotics at least once.

### 3.3 Hematology and immune cells in the blood

With the exception of sampling day and some interactions between sampling day and main factors, no other significant differences were found in the hematological profile (Supplementary Table S6). The result session only includes results of FACS analyses of batch 2 due to problems with delivery and unavailability of antibodies to perform the analyses of samples belonging to batch 1.

CC calves had lower numbers of CD45 cells/ml compared to DF calves (*p* ≤ 0.05; Supplementary Table S7). Most of the other blood variables were affected by sampling day (Supplementary Tables S6, S7) and, leukocytes, CD8 + T cells, gamma delta T cells, CD4 + T cells, and NK cells generally decreasing with age (Supplementary Table S7).

### 3.4 Immune parameters in BALF

Calves receiving AB had a higher number of leukocytes in the BALF (11.8 × 10^6^/mL vs. 8.9 × 10^6^/mL) compared to calves not receiving AB on both day 21 and 45 of the experiment (*p* = 0.01; Table 3).

**Table 3:** Effects of source (direct transport from the dairy farms (DF) and subsequent separate housing per dairy farm or transport via a collection center (CC) and subsequent mixed housing) and the prophylactic application of an antibiotic treatment in early-life (AB; yes or no) on broncho-alveolar lavage fluid (BALF) variables of calves measured on day 21 and 45 of the experiment (values are reported as raw means and their pooled SEM^1^).

Parameter	Dairy farm (DF)		Collection center (CC)		SEM	*p*-value						
	No AB	AB	No AB	AB		Source	AB	Batch	Day	Source × AB	Source × Day	AB × Day
Cells (×10^6^/mL)						0.01	<0.01	0.03	0.07	0.50	0.02	0.94
Day 21	5.2	2.1	13.6	18.7	2.7							
Day 45	7.6	11.8	8.9	13.7	3.8							
Alveolar macrophages (%)^2^						<0.01	<0.01	<0.01	0.16	0.84	-	-
Day 21	44.7	60.8	24.7	46.8	4.8							
Day 45	42.3	51.1	30.6	33.9	6.1							
Phagocytosis activity of cells^3^						0.53	0.38	<0.01	<0.01	0.16	0.68	0.84
Day 21	1,024,080	867,403	928,628	783,090	100,249							
Day 45	371,605	447,787	398,295	399,626	97,986							
ROS activity^3^						0.31	0.84	<0.01	<0.01	0.06	0.25	0.77
Day 21	486,302	361,098	553,981	326,974	81,334							
Day 45	1,908,258	1,969,430	1,507,222	773,454	425,555							
Stimulated ROS activity^3^						0.47	0.46	<0.01	<0.01	0.19	0.29	0.43
Day 21	668,806	534,379	723,320	501,483	134,065							
Day 45	1,783,432	1,871,885	1,566,642	728,345	407,251							

1 SEM, standard error of the mean.

2 The model did not converge with the inclusion of the last three interactions, therefore the missing P-values.

3 Expressed as relative fluorescent units (RFU).

Calves that received AB had a higher percentage of alveolar macrophages compared to calves not receiving AB (*p* < 0.01). CC calves had a lower percentage of alveolar macrophages in the BALF compared to DF calves (*p* < 0.01).

### 3.5 Microbiota

**Figure 2:**
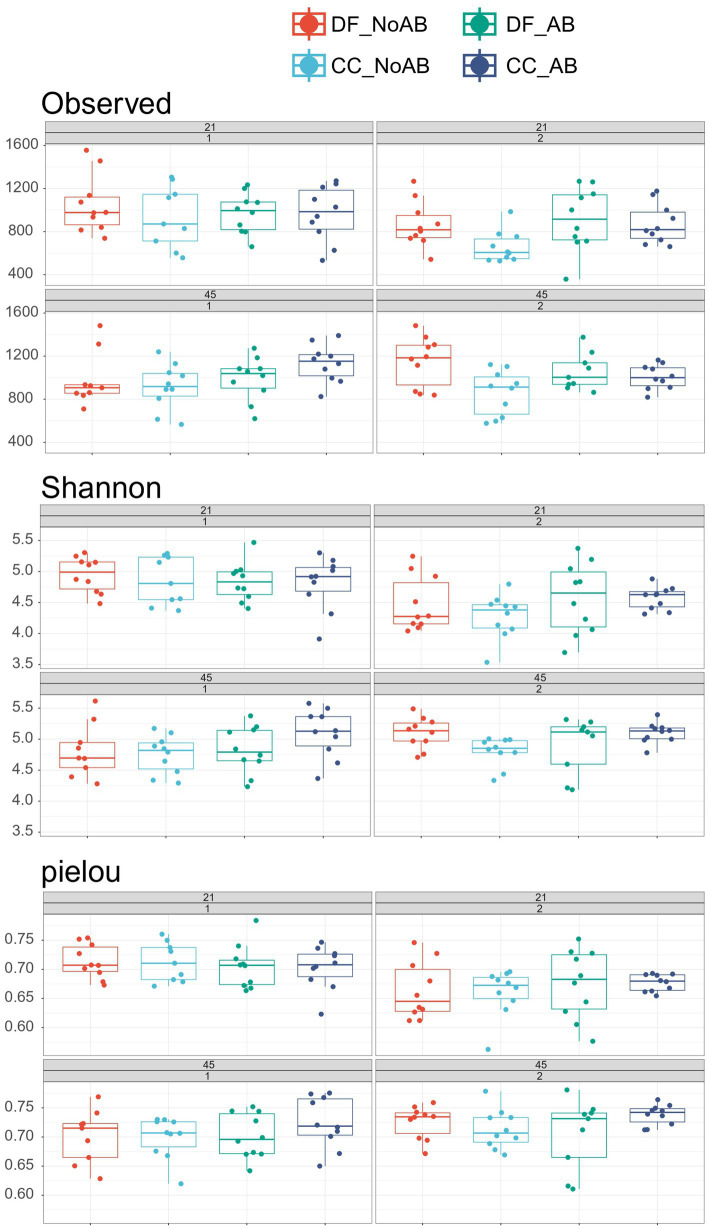
Alpha-diversity measures including Richness (observed species; Source × AB *p*-value = 0.09), Shannon index (Source × AB *p*-value = 0.10), and Pielou’s evenness (Source × AB *p*-value = 0.21) of rectal swabs collected on day 21 and 45 of the experiment. The treatment groups comprehended the following: DF_No AB = calves transported directly from a dairy farm to the experimental farm and not receiving a prophylactic group antibiotic treatment in the first week after arrival; DF_AB = calves transported directly from a dairy farm to the experimental farm, receiving a prophylactic group antibiotic treatment in the first week after arrival; CC_No AB = calves transported first from a dairy farm to a collection center (where they were mixed with other calves), then from the collection center to the experimental farm; not receiving a prophylactic group antibiotic treatment in the first week after arrival; CC_AB = calves transported first from a dairy farm to a collection center (where they were mixed with other calves), then from the collection center to the experimental farm; receiving a prophylactic group antibiotic treatment in the first week after arrival. In all figures results of both batches of calves are shown.

**Figure 3:**
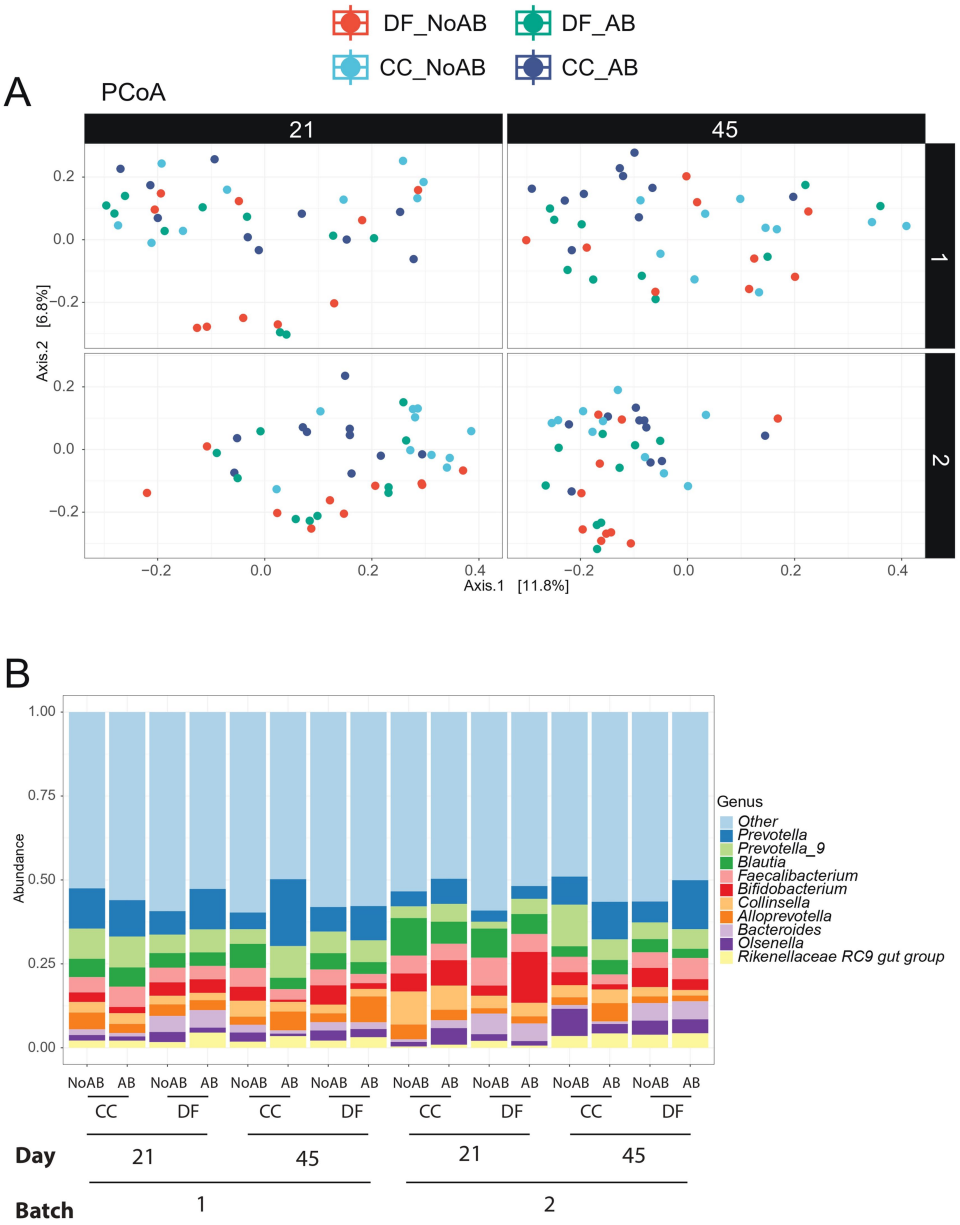
Beta-diversity measures in rectal swabs collected on day 21 and 45 of the experiment. **(A)** PCoA Bray–Curtis dissimilarity (Batch×Day×Source×AB *p*-value <0.01; *p*-values calculated by adonis2); **(B)** Microbiota composition at species level showing the top 10 species in rectal swabs collected on day 21 and 45 of the experiment. The treatment groups comprehended the following: DF_No AB = calves transported directly from a dairy farm to the experimental farm and not receiving a prophylactic group antibiotic treatment in the first week after arrival; DF_AB = calves transported directly from a dairy farm to the experimental farm, receiving a prophylactic group antibiotic treatment in the first week after arrival; CC_No AB = calves transported first from a dairy farm to a collection center (where they were mixed with other calves), then from the collection center to the experimental farm; not receiving a prophylactic group antibiotic treatment in the first week after arrival; CC_AB = calves transported first from a dairy farm to a collection center (where they were mixed with other calves), then from the collection center to the experimental farm; receiving a prophylactic group antibiotic treatment in the first week after arrival. In all figures results of both batches of calves are shown.

A tendency for Source × AB interaction (*p* = 0.10; Table 4; Figure 2) was found for the microbial richness and Shannon index in fecal samples, which were lowest in CC_No AB calves. The results of the principle coordinate analysis (PCoA) revealed a significant interaction between source, AB, batch, and day for fecal microbiota (*p* < 0.01; Figure 3A). Microbiota composition at species level is shown in Figure 3B. Regarding the beta diversity, CC_No AB calves had the lowest percentage of *Subdoligranulum* (Source × AB interaction *p* = 0.01) and *Eubacterium* (Source × AB interaction *p* = 0.04) on both day 21 and 45 compared to all the other calves. DF calves had a lower percentage of *Prevotella* 9 (*p* = 0.03), *Blautia* (*p* = 0.03), and *Phascolarctobacterium* (*p* < 0.01), and a higher percentage of *Bifidobacterium* (*p* = 0.02), *Bacteroides* (*p* = 0.01), *Muribaculaceae* (*p* < 0.01), and *Parabacteroides* (*p* < 0.01) compared to CC calves on both day 21 and 45. Calves receiving AB had a higher percentage of *Muribaculaceae* than calves not receiving AB (*p* = 0.03).

**Table 4:** Effects of source (direct transport from the dairy farms (DF) and subsequent separate housing per dairy farm or transport via a collection center (CC) and subsequent mixed housing) and the prophylactic application of an antibiotic treatment in early-life (AB; yes or no) on fecal microbial alpha diversity indices of calves measured on day 21 and 45 of the experiment (values are reported as raw means and their pooled SEM^1^).

Parameter	Dairy farm (DF)		Collection center (CC)		SEM	*p*-value						
	No AB	AB	No AB	AB		Source	AB	Batch	Day	Source × AB	Source × Day	AB × Day
Richness						0.25	0.28	0.34	<0.01	0.09	0.51	0.95
Day 21	967.6	942.4	772.6	918.4	55.8							
Day 45	1052.4	1020.7	866.5	1070.7	46.7							
Shannon index						0.79	0.41	0.18	<0.01	0.10	0.31	0.65
Day 21	4.7	4.7	4.5	4.7	0.09							
Day 45	4.9	4.9	4.8	5.1	0.07							
Evenness						0.56	0.60	0.20	<0.01	0.21	0.31	0.66
Day 21	0.692	0.690	0.685	0.690	0.009							
Day 45	0.712	0.703	0.714	0.731	0.008							

1 SEM, standard error of the mean.

**Figure 4:**
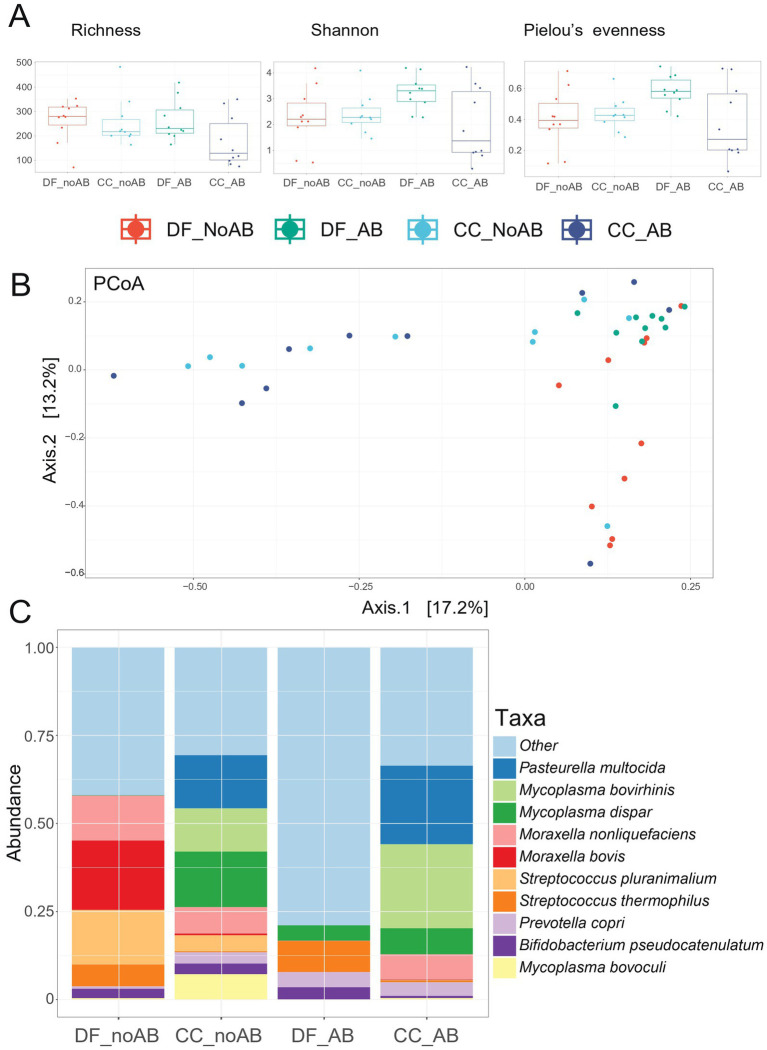
Microbiota analyses of nasal swabs collected on day 7 of the experiment. **(A)** Alpha-diversity measures, including richness (observed species), Shannon index, and Pielou’s evenness (all *p*-values <0.05, calculated by linear modelling); **(B)** Beta-diversity measures, including PCoA Bray–Curtis dissimilarity (AB *p*-value = 0.01; Source *p*-value <0.01; *p-values* calculated by adonis2); **(C)** Microbiota composition at species level showing the top 10 species. These figures concern only batch 2 of calves. The treatment groups comprehended the following: DF_No AB = calves transported directly from a dairy farm to the experimental farm and not receiving a prophylactic group antibiotic treatment in the first week after arrival; DF_AB = calves transported directly from a dairy farm to the experimental farm, receiving a prophylactic group antibiotic treatment in the first week after arrival; CC_No AB = calves transported first from a dairy farm to a collection center (where they were mixed with other calves), then from the collection center to the experimental farm; not receiving a prophylactic group antibiotic treatment in the first week after arrival; CC_AB = calves transported first from a dairy farm to a collection center (where they were mixed with other calves), then from the collection center to the experimental farm; receiving a prophylactic group antibiotic treatment in the first week after arrival.

Microbial richness and Shannon index did not differ in jejunal digesta (Supplementary Figure S2A), but PCoA showed effects of AB and source on jejunal microbiota diversity (*p* < 0.01; Supplementary Figure S2B). Regarding nasal swabs, CC_AB group had the lowest Shannon index and Pielou’s evenness compared to all the other treatment groups (*p* < 0.05; Figure 4A). PCoA showed an antibiotic effect (*p* = 0.01) and a source effect (*p* < 0.01) on microbiota (Figure 4B). CC calves had a higher prevalence of *Mycoplasma bovirhinis, Mycoplasma dispar,* and *Pasteurella multocida* compared to DF calves (species summed: 20–30% vs. 1–2%, see Figure 4C).

### 3.6 Post-mortem examinations

The CC calves had a higher lung/heart ratio compared to DF calves (2.59 vs. 2.29; *p* < 0.01). A higher proportion of calves from a CC had abnormalities in the apical lung lobe (28 vs. 4 calves; *p* < 0.01) and a higher total lung score (34 vs. 5 calves; *p* = 0.04) compared to DF calves. Presence of enlarged tracheal lymph nodes, pus in the trachea, and pus and mucus in the bronchi did not differ among treatments.

## 4 Discussion

The current experiment was designed as a proof of concept study under controlled conditions, with the aim to investigate the effects of two microbial challenges relevant to current veal production on the gut microbiota, immune responses in the blood and lung fluid, and clinical health of veal calves. A range of detailed animal-based measurements was recorded; hence the number of animals included was relatively low in comparison with large-scale studies in practice [e.g., Marcato et al. (4, 19)]. Correspondingly, the housing conditions of the CC calves in our controlled experiment differed from those on veal farms in practice, where much larger numbers of calves are mixed and housed within a stable. Moreover, the factor “source” in our study represents a composite exposure (transport duration, mixing, stress, and pathogen exposure), limiting the ability to pinpoint which specific component is responsible for the observed effects. Overall, this proof of concept study provided highly promising results, despite our limitations to fully control for calf background (e.g., dairy farm of origin) in our experimental design. It should be noted that the dairy farms of origin from which calves were transported directly to the experimental facilities were standard commercial farms with average calf-rearing conditions. Hence, there is no *a priori* reason to assume that the rearing conditions of calves transported directly from the dairy farm differed from those of calves transported from the collection center, which also originated from regular Dutch dairy farms. Despite these limitations and the smaller sample size compared to practice, this study showed that direct transportation of calves from the dairy farms and subsequent housing per DF origin, positively affects calf health. Calves originating from a collection center and not receiving prophylactic antibiotics had the lowest species number in the fecal microbiota measured on day 21, the lowest Shannon Index on day 45, the lowest percentage of alveolar macrophages, the highest prevalence of clinical problems, and the lowest ADG throughout the trial. Transportation via a collection center, which involves mixing calves from different dairy farms and, therefore, exposure to pathogens against which they may lack antibodies, is generally considered an important risk factor for health problems in (surplus) calves (4, 23, 24). Surplus calves frequently experience failure of passive transfer (FTPI) and thus they are at greater risk for infection in an environment with high infection pressure (25, 26). Although no significant differences in clinical health were observed upon arrival between calves transported directly from dairy farms and those transported via the collection center, the mixing of animals from different sources at the collection center, combined with the absence of antibiotics, likely contributed to the higher prevalence of clinical issues observed in the subsequent period of this experiment. Moreover, these clinical problems observed in the current trial, i.e., coughing, nasal discharge, fever, presence of loose and liquid manure, and sunken eyes, align with symptoms commonly reported in other studies in the first weeks post-transport at the veal farm (4, 26–28). A number of recent studies clearly indicate that (respiratory) health problems in calves are associated with reduced growth performance. For example, the presence of lung lesions observed in live animals with the use of thoracic ultrasonography (TUS), which is indicative of BRD, corresponded with a decreased ADG and a lower carcass weight (29–32). Marcato et al. (19) found that calves that received three or more individual treatments with antibiotics or other medicines during the fattening cycle had a much lower carcass weight in comparison with untreated calves or calves treated only once, providing more indirect support for the relationship between health and growth performance in calves. In line with these studies, our results showed that CC_No AB had a reduced ADG during the first 4 weeks of the experiment, and a tendency for a reduced ADG throughout the observational period compared to calves directly transported from the dairy farms, regardless of treatment with antibiotics.

In addition, CC calves and calves not receiving AB presented less alveolar macrophages in BALF samples compared to the other animals. Alveolar macrophages are resident macrophages in the airways and lungs, where they serve as the primary immune sentinels of the respiratory tract. They play an important role in scavenging microbes such as viruses, bacteria, and fungi, inhaled environmental particles like coal, silica, asbestos, tissue debris, and cancer cells (33). It has been proposed that, due to repeated environmental challenges such as recurrent infections, resident alveolar macrophages decrease (34). Thus the lower percentage of alveolar macrophages in the lungs of CC calves and those without AB might reflect depletion due to ongoing (respiratory tract) infections (as seen also from our clinical health observations). This hypothesis is not yet confirmed, thus further study of this potential mechanism would require measuring numbers of alveolar macrophages at multiple timepoints in combination with functional analysis, by monocyte derived macrophages.

To our knowledge, this is one of the first studies comparing fecal microbiota between veal calves transported directly from dairy farms and those via a collection center. Previous research in beef calves shows that the fecal microbiota can vary significantly depending on facility, farm, or location of birth and raising (35, 36). Thus, management practices of calves should always be carefully considered as factors shaping gut microbial communities alongside diet, administration of antimicrobials, genetics, breed, and age of the animal (37). Few studies (38, 39) have investigated the therapeutic oxytetracycline effects on calf fecal microbiota. Despite the small samples size, Oultram et al. (38) reported differences in the prevalence of the genus *Lactobacillus* and increased species richness in the treated calves compared to controls. This is in line with our observations of lower species richness and lower Shannon diversity index of fecal microbiota found in CC_No AB calves, potentially associated with gut inflammation and disease (40).

Regarding fecal microbial beta-diversity, several studies (35, 37, 41, 42) investigated antibiotic impact on the gut microbiota. Other studies investigated the impact of transportation, as a major stress factor, on both respiratory and gut microbiota (43–46). However, none of the previous studies have combined antibiotic treatment with calf source. In the current study CC calves had a higher percentage of *Prevotella* 9, *Blautia*, and *Phascolarctobacterium*, and a lower percentage of *Bifidobacterium*, *Bacteroides*, *Muribaculaceae*, and *Parabacteroides* compared to calves transported via DF. In particular, CC_No AB calves showed the lowest percentage of *Subdoligranulum* and *Eubacterium,* both considered beneficial genera (47, 48), compared to all the other calves. This finding suggests that transporting calves via a collection center and refraining from subsequent prophylactic AB treatment might negatively impact the gut microbiome of calves. It could be speculated that the higher percentage of more pathogenic bacterial species with CC calves indicates the out competition of more beneficial species, especially in absence of AB, resulting in the lower percentage of *Subdoligranulum* and *Eubacterium* for CC_No AB calves. Calves receiving AB had a higher percentage of *Muribaculaceae* compared to calves not receiving AB. Previous studies reported that *Bifidobacterium* can prevent gastrointestinal infections in calves (40) and contribute to gut health by production of inhibitory substances (49), and modulating the gastrointestinal immune system response (40, 50). *Muribaculaceae* is a common and abundant symbiotic bacteria family in the gut, specialized in fermenting complex carbohydrates (51) and linked to longevity (52) and together with *Bacteroides* negatively associated with diarrhea in calves (53). Fan et al. (54) found decreased relative abundance of *Parabacteroides* and increased *Phascolarctobacterium* in calves with abnormal fecal consistency. According to these studies, our results suggest that direct transport from the dairy farm is beneficial for growth of beneficial gut bacteria compared to transport via a collection center. Moreover, Chen et al. (53) reported that *Prevotella* 9 might help to prevent calf diarrhea and Zhang et al. (55) suggested that *Blautia* is a genus with potential probiotic components in calves. However, these species were lower in calves transported directly from the dairy farm compared to those via a collection centers, contrasting other findings. Jejunal samples from batch 2 were analyzed because the fecal microbiota of these calves showed major changes. The purpose of analyzing these additional gut samples was to gain further insight into the small intestinal microbiota and to investigate whether the results of the jejunal digesta could be comparable to those of fecal samples. In other words, we wanted to establish whether the microbiome of more proximal segments, such as the jejunum, could resemble the fecal microbiota. Jejunal microbiota composition measured in these samples was not affected by the treatments. This implies that the link between proximal part of the gut and the fecal microbiota is complex and it might be that changes in the first part of the gut can be visible but at another timepoint. However, this would require further study.

Nasal swabs collected on day 7 were also analyzed only for batch 2. Despite the limitation in sample size and sampling moment (as reference samples on a different time are not available), the nasal samples on day 7 showed a higher abundance of *Mycoplasma* and *Pasteurella multocida* in calves from the collection center relative to calves transported directly from dairy farms. These two bacteria are mainly associated with the onset of respiratory diseases (56) and their higher presence in calves from the collection center at the beginning of the experiment aligns with the higher prevalence of clinical problems and poorer performance observed in these calves at subsequent time points. Thus, these results indicate that calves passing through a collection center and being mixed with animals originating from other dairy farms might be more exposed to pathogens and more prone to diseases than calves directly transported to the veal farm and not being mixed.

Post-mortem lung examination showed that more CC calves had a high lung-to-heart weight ratio, more lung abnormalities and a higher (and thus worse) lung score compared to DF calves. This suggests that CC calves may have experienced respiratory infections. The lung-to-heart ratio is a measure reflecting respiratory health in calves, and showing how much gas-exchange surface (lungs) is available relative to the blood-pumping capacity (heart). The size of heart and lungs need to be proportionate so that one is not chronically limiting the other (57).

The heart-to-lung weight ratio is often used as an indicator of the degree of inflammatory infiltrates in the lungs of calves (58). Lungs with less consolidation and thus with fewer infiltrates weigh less and have a smaller lung-to-heart ratio than lungs with more severe infiltrates (58). Presence of purulent material and other lung lobe abnormalities is also a sign of chronic respiratory infections in veal calves (5, 59).

The use of group antibiotic treatments represents a major concern in the current veal sector. Previous studies (19, 60, 61) have shown that an average of at least three group treatments are used, mainly during the first weeks post-transport to counteract the occurrence of mainly respiratory and gastrointestinal diseases. In the current experiment a combination treatment of antibiotics and aspirin was used in compliance with regular health management protocols. However, this combination made the distinction between antimicrobial effects and anti-inflammatory or immunomodulatory effects difficult, in particular with regard to their impact on the microbiota and immune outcomes. With regard to antibiotics we used oxytetracycline, which is a commonly used oral antibiotic for calves, and it was chosen due to its mechanism of action against lung and gut infections. It was expected to impact both the gut and lung microbiota, and this study showed that administration of group antibiotic treatment was especially necessary for the calves transported via a collection center. Calves transported directly from dairy farms, regardless of receiving antibiotics, performed similarly. As previously mentioned, transporting calves via a collection center is challenging, and the withholding antibiotic treatment in calves transported via collection center reduced health and growth parameters compared to all the other treatment groups. Although not statistically different from the other treatments, 10 out of 24 CC_No AB calves required individual antibiotic treatment, due to clinical signs of illness. Thus, in terms of the general resilience of calves, the current results suggest that direct transportation from the dairy farms and subsequent separate housing improves calf health. Adding an additional step via a collection center and mixing of calves increase health challenges not only in the first days post-transport but also in the longer term (as confirmed by post-mortem lung inspection). Direct transportation without mixing calves will likely reduce the requirement for antibiotics, which could contribute to increase the sustainability of the sector.

Overall, elements of the concept tested in the present experiment clearly need to be examined under practical conditions. In addition to confirming the present findings, numerous aspects need to be studied and questions to be answered, before transporting calves directly from dairy farms to veal farms could be feasibly implemented in practice. These include logistical challenges, housing adaptations, receiving protocols for incoming calves, and optimal batch/group size composition, among others.

## 5 Conclusion

The number of animals included in this study was relatively low in comparison with large-scale studies in practice. However, the current proof of concept study showed that transporting calves via a collection center and subsequently housing them in mixed groups without prophylactically administered antibiotics contributed to a higher prevalence of clinical problems and lung abnormalities, a lower performance, less beneficial microbiota in fecal and nasal samples, and a lower number of alveolar macrophages in calves during the trial. Direct transportation of calves from the dairy farms of origin to their destination, without mixing animals from different sources and with subsequent housing in separate rooms, seemed to be the most ideal scenario for the calves in this experiment. This approach could form the basis for developing and implementing alternative calf sourcing and rearing strategies in the veal calf production chain. Antibiotic use can partly compensate for the difference in health challenges between CC and DF calves. Therefore, alternative routing of calves (and/or prevention of mixing animals from different sources) could be an important preventive measure to reduce antibiotic use in veal production.

## Data Availability

The microbiota data can be found here: European Nucleotide Archive (ENA) under accession number PRJEB108096. Further inquiries can be directed to the corresponding author.

## Footnotes

1
https://github.com/gbouras13/coatofarms

## Supplementary material

SUPPLEMENTARY FIGURE S1 Prevalence of clinically ill calves during the experiment. **(A)** Loose and liquid manure; **(B)** Coughing; **(C)** Eye discharge; **(D)** Sunken eyes; **(E)** Nasal discharge; **(F)** Fever. The treatment groups comprehended the following: DF_No AB= calves transported directly from a dairy farm to the experimental farm and not receiving a prophylactic group antibiotic treatment in the first week after arrival; DF_AB= calves transported directly from a dairy farm to the experimental farm, receiving a prophylactic group antibiotic treatment in the first week after arrival; CC_No AB= calves transported first from a dairy farm to a collection center (where they were mixed with other calves), then from the collection center to the experimental farm; not receiving a prophylactic group antibiotic treatment in the first week after arrival; CC_AB= calves transported first from a dairy farm to a collection center (where they were mixed with other calves), then from the collection center to the experimental farm; receiving a prophylactic group antibiotic treatment in the first week after arrival.

SUPPLEMENTARY FIGURE S2 Microbiota analyses in jejunum samples collected on day 49 of the experiment. **(A)** Alpha-diversity measures: Richness (observed species), Shannon index, and Pielou's evenness (all *p*-values;0.05, calculated by linear modelling); **(B)** Beta-diversity measure, PCoA Bray-Curtis dissimilarity (*p*-values for AB and Source;0.01; *p*-values calculated by adonis2); **(C)** Microbiota composition at species level showing the top 10 species. These figures concern only batch 2 of calves. The treatment groups comprehended the following: DF_No AB= calves transported directly from a dairy farm to the experimental farm and not receiving a prophylactic group antibiotic treatment in the first week after arrival; DF_AB= calves transported directly from a dairy farm to the experimental farm, receiving a prophylactic group antibiotic treatment in the first week after arrival; CC_No AB= calves transported first from a dairy farm to a collection center (where they were mixed with other calves), then from the collection center to the experimental farm; not receiving a prophylactic group antibiotic treatment in the first week after arrival; CC_AB= calves transported first from a dairy farm to a collection center (where they were mixed with other calves), then from the collection center to the experimental farm; receiving a prophylactic group antibiotic treatment in the first week after arrival.
