# Supplementary figures and images for: Impact of transport source (dairy farms vs. collection center) and post-arrival housing in combination with prophylactic antibiotic treatment on performance, lung health and microbiota of veal calves
Source: Front Vet Sci. 2026 Mar 2;13:1715667. doi: 10.3389/fvets.2026.1715667 (PMC12989376; doi:10.3389/fvets.2026.1715667)

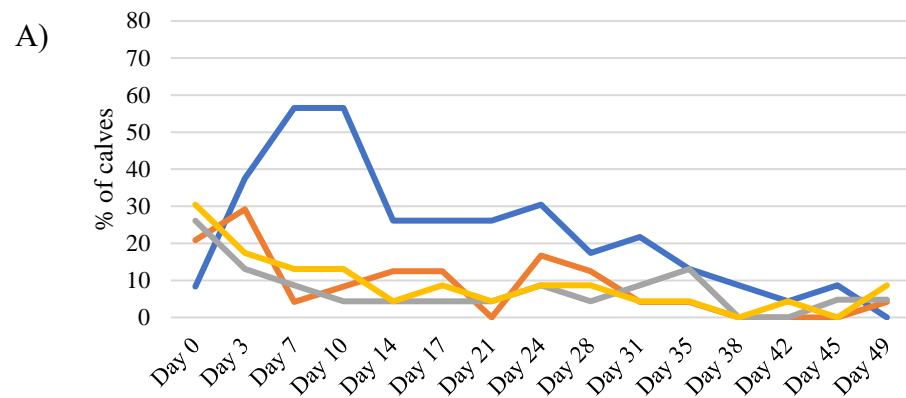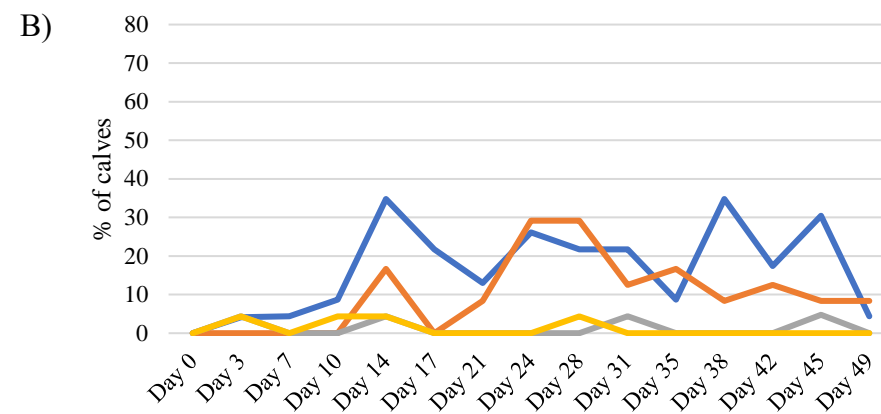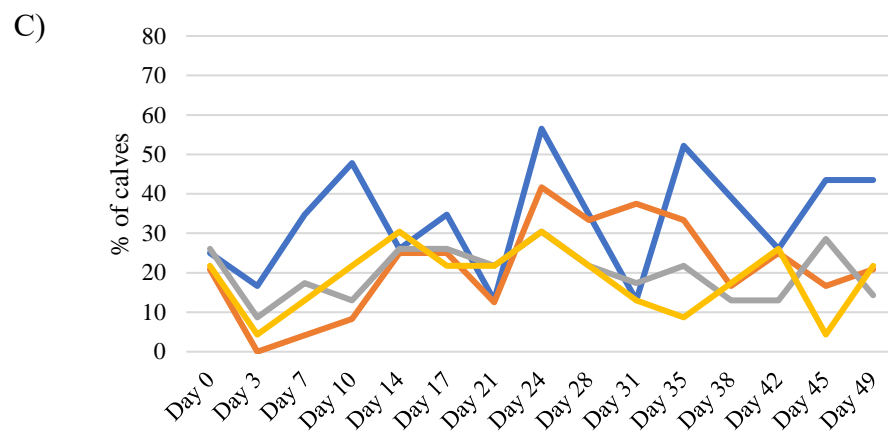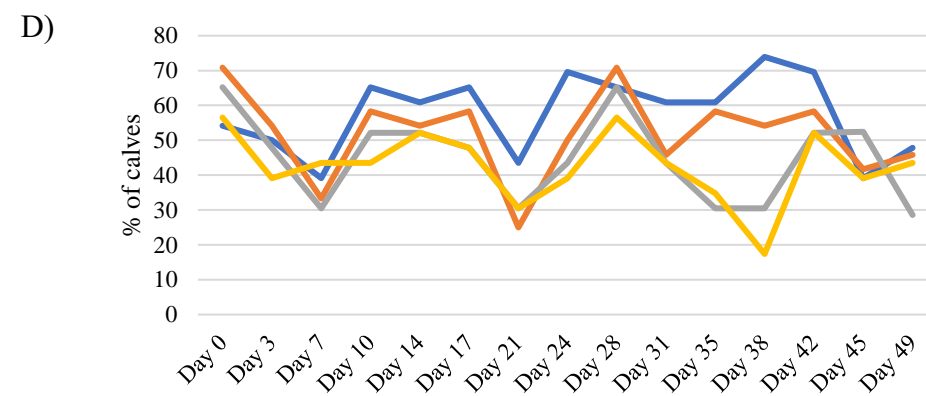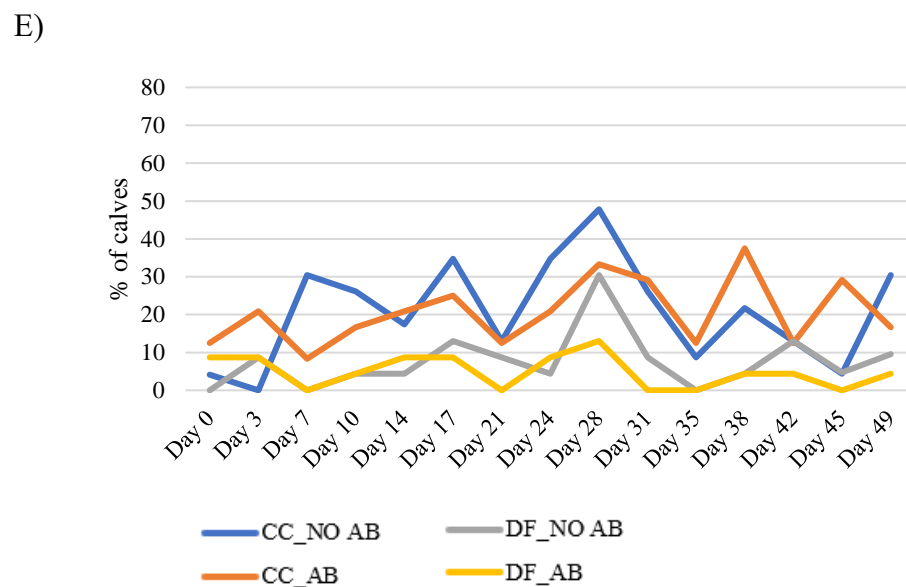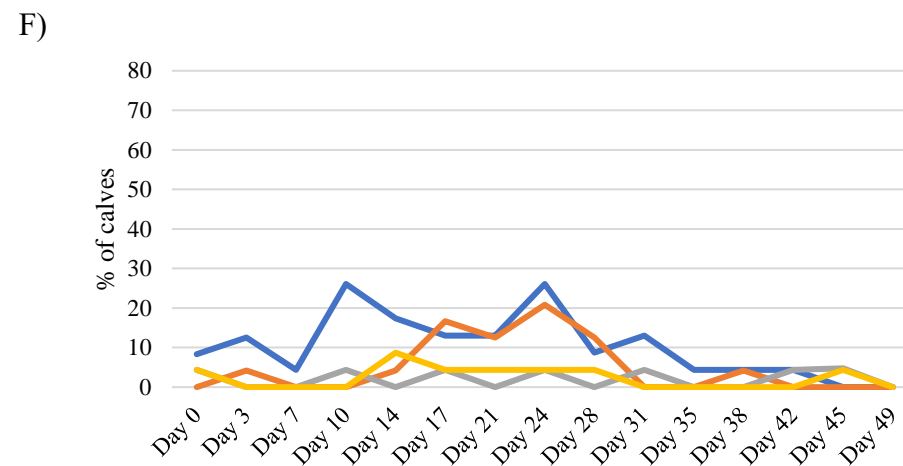

Supplement: SUPPLEMENTARY FIGURE S1 — Prevalence of clinically ill calves during the experiment. (A) Loose and liquid manure; (B) Coughing; (C) Eye discharge; (D) Sunken eyes; (E) Nasal discharge; (F) Fever. The treatment groups comprehended the following: DF_No AB= calves transported directly from a dairy farm to the experimental farm and not receiving a prophylactic group antibiotic treatment in the first week after arrival; DF_AB= calves transported directly from a dairy farm to the experimental farm, receiving a prophylactic group antibiotic treatment in the first week after arrival; CC_No AB= calves transported first from a dairy farm to a collection center (where they were mixed with other calves), then from the collection center to the experimental farm; not receiving a prophylactic group antibiotic treatment in the first week after arrival; CC_AB= calves transported first from a dairy farm to a collection center (where they were mixed with other calves), then from the collection center to the experimental farm; receiving a prophylactic group antibiotic treatment in the first week after arrival. [file Image_1.pdf]

A

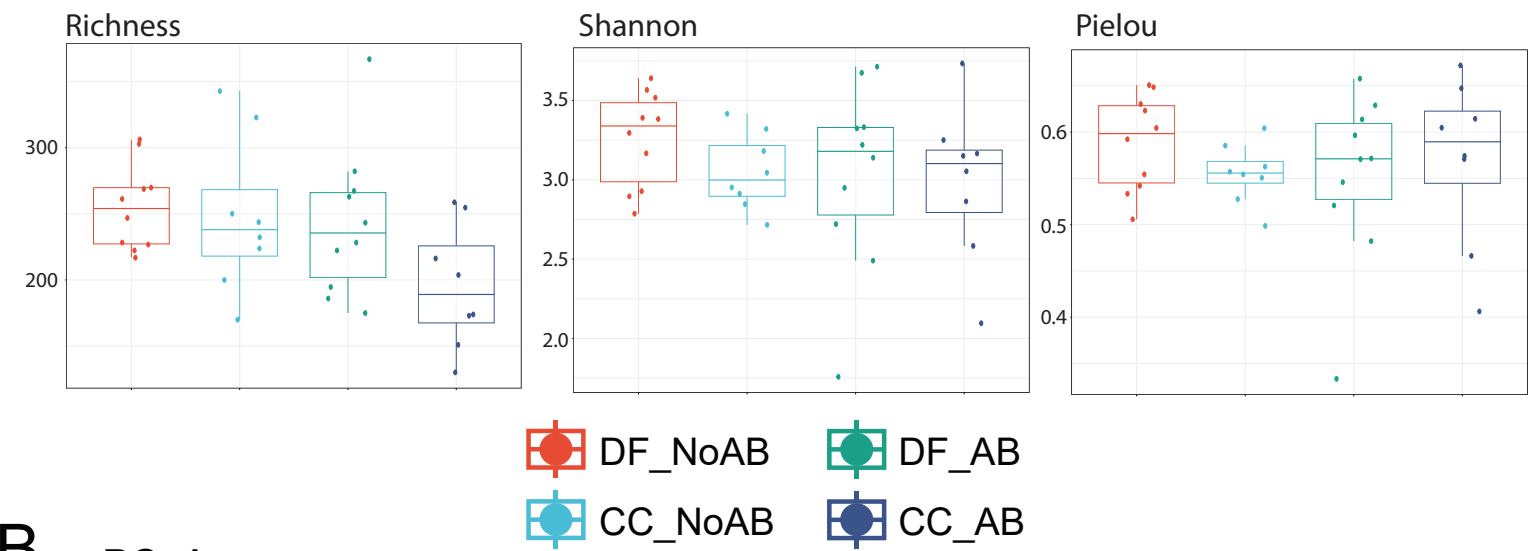

B

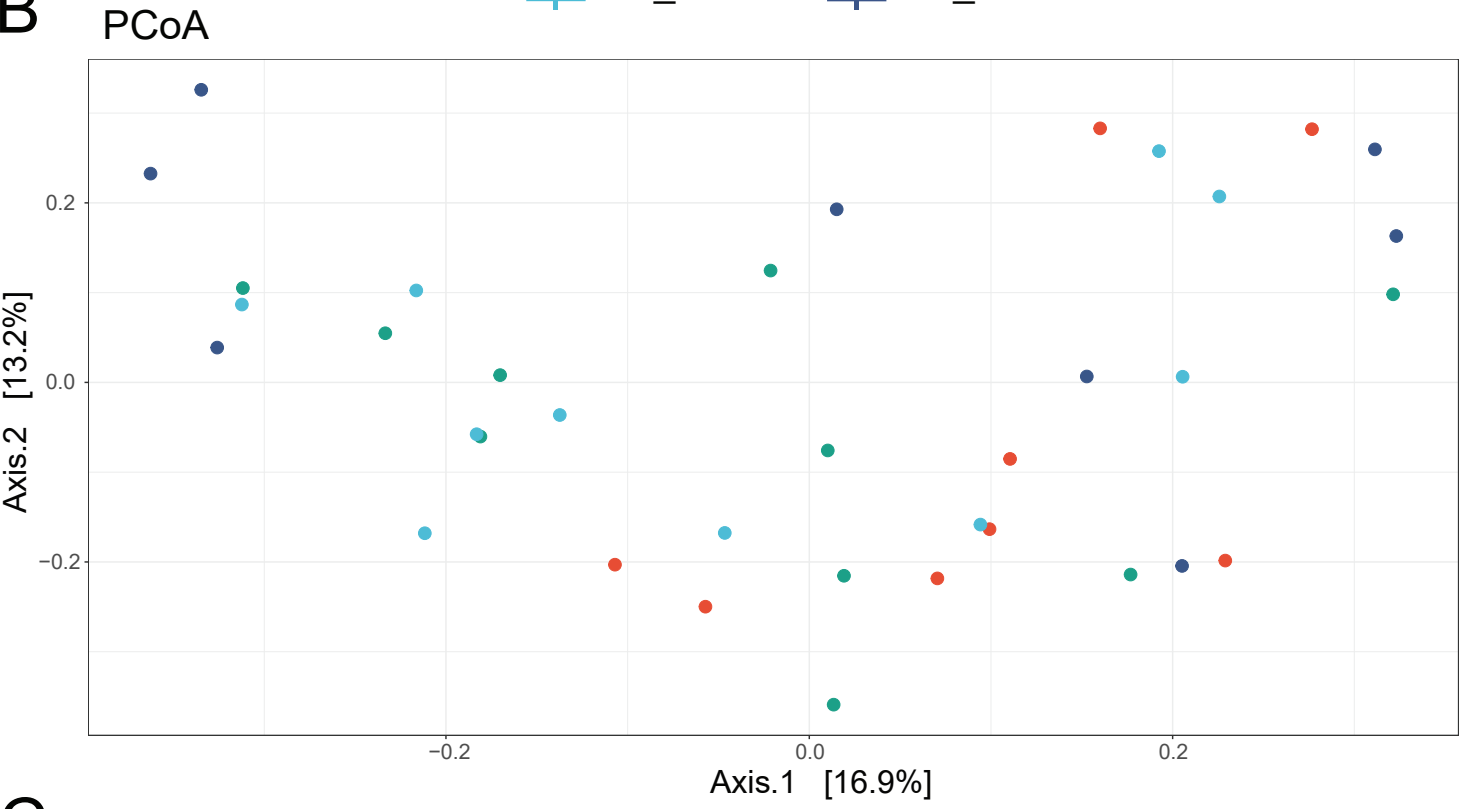

C

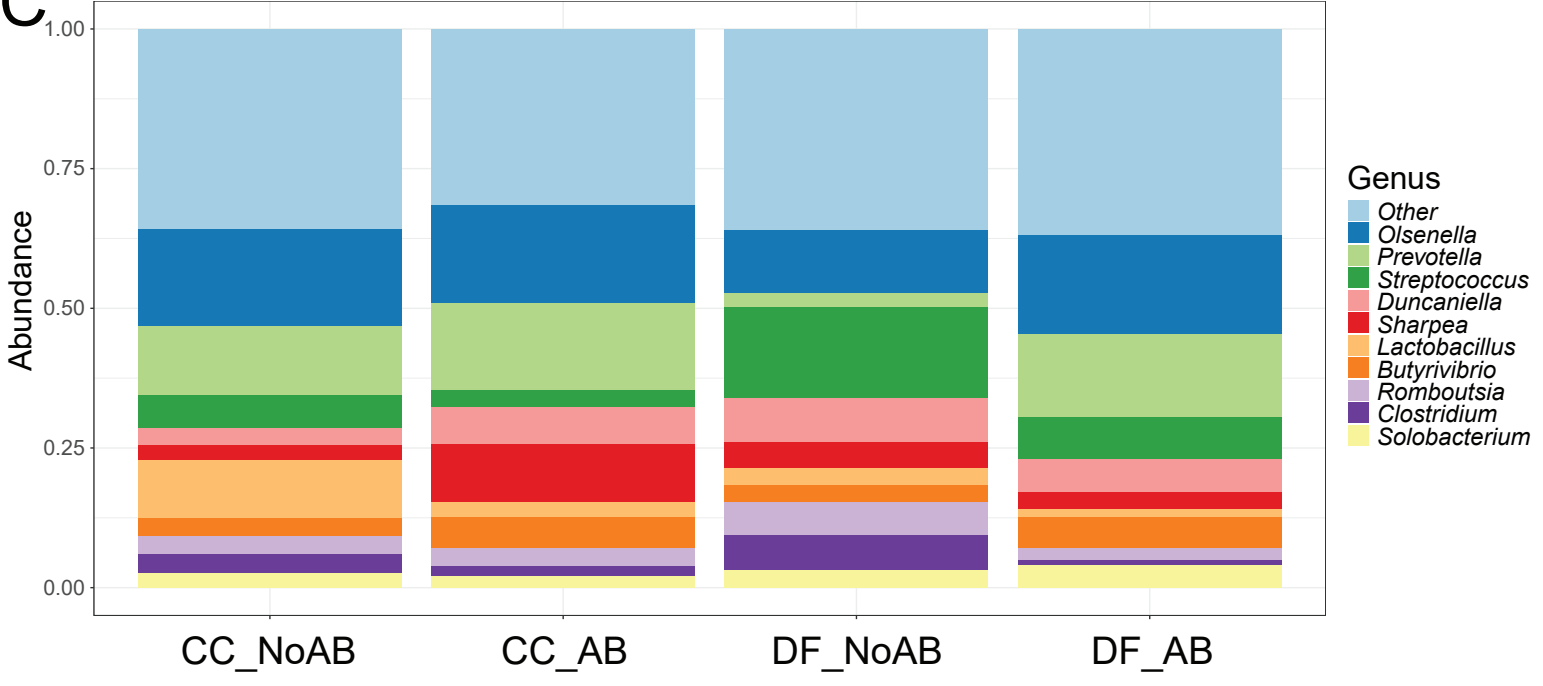

Supplement: SUPPLEMENTARY FIGURE S2 — Microbiota analyses in jejunum samples collected on day 49 of the experiment. (A) Alpha-diversity measures: Richness (observed species), Shannon index, and Pielou's evenness (all p-values;0.05, calculated by linear modelling); (B) Beta-diversity measure, PCoA Bray-Curtis dissimilarity (p-values for AB and Source;0.01; p-values calculated by adonis2); (C) Microbiota composition at species level showing the top 10 species. These figures concern only batch 2 of calves. The treatment groups comprehended the following: DF_No AB= calves transported directly from a dairy farm to the experimental farm and not receiving a prophylactic group antibiotic treatment in the first week after arrival; DF_AB= calves transported directly from a dairy farm to the experimental farm, receiving a prophylactic group antibiotic treatment in the first week after arrival; CC_No AB= calves transported first from a dairy farm to a collection center (where they were mixed with other calves), then from the collection center to the experimental farm; not receiving a prophylactic group antibiotic treatment in the first week after arrival; CC_AB= calves transported first from a dairy farm to a collection center (where they were mixed with other calves), then from the collection center to the experimental farm; receiving a prophylactic group antibiotic treatment in the first week after arrival. [file Image_2.pdf]
