# Supplementary material for: Impact of transport source (dairy farms vs. collection center) and post-arrival housing in combination with prophylactic antibiotic treatment on performance, lung health and microbiota of veal calves
Source: Front Vet Sci. 2026 Mar 2;13:1715667. doi: 10.3389/fvets.2026.1715667 (PMC12989376; doi:10.3389/fvets.2026.1715667)
Supplement: Supplementary file 3 [file Table_1.docx]

**Supplementary Table 1.** Practical feeding schedule used during the calf experiment.

| Experimental week | Milk replacer^1^ (g/calf/day) | Reconstituted milk replacer concentration (g/L) | Concentrates^2^ (g/calf/day) | Chopped straw  (g/calf/day) |
| --- | --- | --- | --- | --- |
| 1 | 280-305 | 140-153 | 40 | 4 |
| 2 | 310-400 | 141-148 | 70 | 7 |
| 3 | 415-475 | 153-141 | 100 | 10 |
| 4 | 485-500 | 147-132 | 150 | 15 |
| 5 | 502-518 | 132-130 | 200 | 20 |
| 6 | 524-530 | 131-133 | 250 | 25 |
| 7 | 532-540 | 133-135 | 300 | 30 |
| 8 | 540 | 135 | 350 | 35 |
| ^1^ Denkaveal starter in the first 2 weeks and after Gold milk replacer of Denkavit B.V. (Voorthuizen, the Netherlands)  ^2^ Denkaveal Mueslibar Extra of Denkavit B.V. (Voorthuizen, the Netherlands) | | | | |

**Supplementary Table 2.** Milk replacer and pelleted nutrient composition provided to calves during the experiment.

|  | | Milk replacer^1^ | Concentrates^2^ |
| --- | --- | --- | --- |
| Carbohydrates | 44.8% | | 66.2% |
| Crude protein | 23.9% | | 17.0% |
| Crude fat | 20.7% | | 5.1% |
| Crude fiber | 0.2% | | 5.5% |
| Ash | 8.3% | | 4.8% |
| Calcium (Ca) | 0.76% | | 0.70% |
| Phosphorus (P) | 0.64% | | 0.20% |
| Sodium (Na) | 0.66% | | 0.32% |
| Magnesium (Mg) |  | | 0.20% |

^1^ Denkaveal starter in the first 2 weeks and after Gold milk replacer of Denkavit B.V. (Voorthuizen, the Netherlands)

^2^ Denkaveal Mueslibar Extra of Denkavit B.V. (Voorthuizen, the Netherlands)

**Supplementary Table 3.** Protocol used to assess clinical health of calves at individual level during the experiment.

| **Health parameter** | **Score** | **Explanation** |
| --- | --- | --- |
| Navel inflammation | 0 | No signs of inflammation |
|  | 1 | Swollen, without discharge |
|  | 2 | Swollen, with discharge |
| Joint inflammation | 0 | No evidence of joint problems |
|  | 1 | Slight swelling, not warm or painful |
|  | 2 | Swelling with pain, heat, slight lameness |
| Loose or liquid manure | 0 | No loose or liquid manure |
|  | 1 | Pasty manure |
|  | 2 | Watery manure |
| Coughing | 0 | No coughing |
|  | 1 | Induced single coughing |
|  | 2 | Repeated coughing |
| Eye discharge | 0 | No eye discharge |
|  | 1 | Slight watery discharge |
|  | 2 | Moderate amount of bilateral ocular discharge |
| Sunken eyes | 0 | Normal, bright eyes |
|  | 1 | Eyes markedly recessed into the orbits |
| Ears | 0 | Normal |
|  | 1 | Slight unilateral droop |
|  | 2 | Head tilt or bilateral droop |
| Nasal discharge | 0 | No discharge |
|  | 1 | Watery discharge |
|  | 2 | Purulent discharge |
| Skin elasticity | 0 | Skin tent returns to normal < 2 s |
|  | 1 | Skin tent returns to normal in 2-4 s |
|  | 2 | Skin tent returns to normal in > 8-10 s |

**Supplementary Table 4.** An overview of the monoclonal antibodies and their target that were used in this study.

| **Target** | **Antibody** | **Clone** | **Isotype** |
| --- | --- | --- | --- |
| Leukocytes | Mouse-anti-bovine-CD45-AF647 | CC1 | IgG1 |
| T helper cells (CD4+) | Mouse-anti-bovine CD4-AF700 | CC8 | IgG2a |
| Cytotoxic T cells (CD8+) | Mouse-anti-bovine CD8-PB | CC63 | IgG2a |
| Gamma delta T cells (γδ) | Mouse-anti-bovine WC1-FITC | CC15 | IgG2a |
| Natural killer cells (NK) | Mouse-anti-bovine CD335-PE | AKS1 | IgG1 |
| MHCII | Mouse-anti-bovine MHCII-PE | IL-A21 | IgG2a |
| CD11c | Mouse-anti-bovine CD11c-UNL | BAQ153A | IgM |
| Mouse IgM | Rabbit-anti-mouse-IgM-BV421 |  |  |

**Supplementary Table 5.** Effects of source (direct transport from the dairy farms (DF) and subsequent separate housing per dairy farm or transport via a collection center (CC) and subsequent mixed housing) and batch of calves on prevalence of health problems (expressed as % of calves) upon arrival at the experimental facility (values are reported as raw means and their pooled SEM^1^).

|  | Source | |  |  | Batch | |  |  |
| --- | --- | --- | --- | --- | --- | --- | --- | --- |
| Parameter | CC | DF | SEM | P-value | 1 | 2 | SEM | P-value |
| No. of calves | 48 | 46 |  |  | 48 | 46 |  |  |
| Fever | 8.3 | 4.3 | 3.5 | 0.38 | 10.4 | 2.2 | 3.3 | 0.20 |
| Navel inflammation | 35.4 | 21.7 | 6.5 | 0.20 | 20.8 | 36.9 | 6.5 | 0.17 |
| Loose or liquid manure | 14.6 | 28.3 | 5.9 | 0.35 | 35.4 | 6.5 | 5.3 | 0.05 |
| Eye discharge | 22.9 | 23.9 | 6.2 | 0.96 | 35.4 | 10.9 | 5.8 | 0.02 |
| Sunken eyes | 62.5 | 60.9 | 7.2 | 0.96 | 35.4 | 89.1 | 5.8 | < 0.01 |
| Drooped ears | 18.7 | 21.7 | 6.6 | 0.89 | 31.2 | 4.3 | 4.9 | 0.02 |
| Nasal discharge | 8.3 | 4.3 | 3.5 | 0.58 | 6.2 | 6.2 | 3.6 | 0.97 |

^1^SEM= standard error of the mean.

**Supplementary Table 6.** Effects of source (direct transport from the dairy farms (DF) and subsequent separate housing per dairy farm or transport via a collection center (CC) and subsequent mixed housing) and the prophylactic application of an antibiotic treatment in early-life (AB; yes or no) on blood hematological parameters of calves measured on different days (values are reported as raw means and their pooled SEM^1^).

|  | Dairy farm (DF) | | Collection center (CC) | |  | *P*-value | | | | | | |
| --- | --- | --- | --- | --- | --- | --- | --- | --- | --- | --- | --- | --- |
| Parameters | AB | No AB | AB | No AB | SEM | Source | AB | Batch | Day | Source×AB | Source×Day | AB×Day |
| Hb (mmol/l) |  |  |  |  |  | 0.11 | 0.94 | 0.11 | <0.01 | 0.77 | 0.87 | 0.21 |
| Day 1 | 6.6 | 6.6 | 6.3 | 6.0 | 0.2 |  |  |  |  |  |  |  |
| Day 7 | 6.2 | 6.2 | 5.8 | 6.1 | 0.3 |  |  |  |  |  |  |  |
| Day 21 | 6.7 | 6.9 | 6.4 | 6.6 | 0.2 |  |  |  |  |  |  |  |
| Day 35 | 6.7 | 6.8 | 6.7 | 6.4 | 0.3 |  |  |  |  |  |  |  |
| Day 45 | 5.8 | 5.8 | 5.7 | 5.6 | 0.1 |  |  |  |  |  |  |  |
| Day 51 | 6.5 | 6.2 | 6.3 | 6.0 | 0.1 |  |  |  |  |  |  |  |
| Ht (%) |  |  |  |  |  | 0.10 | 0.95 | 0.16 | <0.01 | 0.93 | 0.40 | 0.29 |
| Day 1 | 33.1 | 32.7 | 30.6 | 29.4 | 1.4 |  |  |  |  |  |  |  |
| Day 7 | 31.2 | 31.8 | 29.3 | 30.8 | 1.3 |  |  |  |  |  |  |  |
| Day 21 | 34.7 | 35.5 | 32.4 | 34.4 | 0.9 |  |  |  |  |  |  |  |
| Day 35 | 34.9 | 35.3 | 34.9 | 33.2 | 1.7 |  |  |  |  |  |  |  |
| Day 45 | 28.6 | 28.7 | 27.9 | 27.6 | 0.6 |  |  |  |  |  |  |  |
| Day 51 | 32.0 | 26.9 | 30.6 | 29.3 | 1.6 |  |  |  |  |  |  |  |
| Mcv (f/l) |  |  |  |  |  | 0.41 | 0.94 | 0.54 | <0.01 | 0.28 | 0.86 | 0.96 |
| Day 1 | 37.5 | 36.6 | 36.3 | 36.8 | 0.5 |  |  |  |  |  |  |  |
| Day 7 | 35.6 | 35.2 | 34.2 | 34.9 | 0.6 |  |  |  |  |  |  |  |
| Day 21 | 35.5 | 34.9 | 34.3 | 34.8 | 0.3 |  |  |  |  |  |  |  |
| Day 35 | 33.3 | 33.0 | 32.7 | 32.9 | 0.4 |  |  |  |  |  |  |  |
| Day 45 | 31.3 | 31.0 | 30.8 | 30.8 | 0.5 |  |  |  |  |  |  |  |
| Day 51 | 31.2 | 30.8 | 30.6 | 31.1 | 0.5 |  |  |  |  |  |  |  |
| Platelets (g/l) |  |  |  |  |  | 0.80 | 0.26 | 0.01 | <0.01 | 0.63 | 0.61 | 0.13 |
| Day 1 | 842.5 | 771.1 | 851.2 | 833.0 | 76.8 |  |  |  |  |  |  |  |
| Day 7 | 854.0 | 857.8 | 871.5 | 784.0 | 71.1 |  |  |  |  |  |  |  |
| Day 21 | 690.6 | 620.8 | 689.0 | 644.2 | 79.9 |  |  |  |  |  |  |  |
| Day 35 | 635.5 | 615.9 | 550.9 | 661.3 | 64.5 |  |  |  |  |  |  |  |
| Day 45 | 515.1 | 487.9 | 446.5 | 477.6 | 26.4 |  |  |  |  |  |  |  |
| Day 51 | 459.6 | 402.9 | 451.1 | 401.2 | 30.5 |  |  |  |  |  |  |  |
| RBC (g/l) |  |  |  |  |  | 0.50 | 0.94 | 0.65 | <0.01 | 0.63 | 0.39 | 0.04 |
| Day 1 | 8.8 | 8.9 | 8.4 | 7.9 | 0.3 |  |  |  |  |  |  |  |
| Day 7 | 8.7 | 9.0 | 8.5 | 8.8 | 0.3 |  |  |  |  |  |  |  |
| Day 21 | 9.8 | 10.2 | 9.4 | 9.9 | 0.2 |  |  |  |  |  |  |  |
| Day 35 | 10.5 | 10.5 | 10.7 | 10.1 | 0.5 |  |  |  |  |  |  |  |
| Day 45 | 9.1 | 9.3 | 9.1 | 8.9 | 0.2 |  |  |  |  |  |  |  |
| Day 51 | 10.4 | 10.2 | 10.4 | 9.7 | 0.2 |  |  |  |  |  |  |  |
| WBC (g/l) |  |  |  |  |  | 0.70 | 0.15 | 0.11 | <0.01 | 0.74 | 0.02 | 0.18 |
| Day 1 | 8.9 | 8.1 | 9.1 | 9.7 | 0.8 |  |  |  |  |  |  |  |
| Day 7 | 8.1 | 8.1 | 7.9 | 8.0 | 0.6 |  |  |  |  |  |  |  |
| Day 21 | 12.1 | 9.0 | 11.0 | 11.6 | 0.9 |  |  |  |  |  |  |  |
| Day 35 | 9.3 | 8.3 | 9.5 | 7.6 | 0.7 |  |  |  |  |  |  |  |
| Day 45 | 7.8 | 7.3 | 6.3 | 6.1 | 0.4 |  |  |  |  |  |  |  |
| Day 51 | 7.1 | 6.2 | 7.5 | 8.6 | 0.9 |  |  |  |  |  |  |  |
| Lymphocytes (%) |  |  |  |  |  | 0.95 | 0.53 | 0.09 | <0.01 | 0.77 | 0.09 | 0.04 |
| Day 1 | 44.3 | 38.7 | 35.8 | 33.8 | 3.3 |  |  |  |  |  |  |  |
| Day 7 | 47.0 | 45.7 | 45.9 | 45.2 | 3.1 |  |  |  |  |  |  |  |
| Day 21 | 33.0 | 34.3 | 34.3 | 31.8 | 4.5 |  |  |  |  |  |  |  |
| Day 35 | 44.6 | 47.7 | 45.3 | 47.0 | 3.3 |  |  |  |  |  |  |  |
| Day 45 | 48.7 | 52.4 | 53.8 | 56.8 | 2.3 |  |  |  |  |  |  |  |
| Day 51 | 50.1 | 55.2 | 51.8 | 55.2 | 2.1 |  |  |  |  |  |  |  |
| Neutrophils (%) |  |  |  |  |  | 0.96 | 0.62 | 0.07 | <0.01 | 0.79 | 0.05 | 0.01 |
| Day 1 | 55.2 | 60.8 | 63.6 | 65.9 | 3.2 |  |  |  |  |  |  |  |
| Day 7 | 52.3 | 53.9 | 53.4 | 54.5 | 3.0 |  |  |  |  |  |  |  |
| Day 21 | 66.7 | 65.0 | 65.4 | 67.5 | 4.4 |  |  |  |  |  |  |  |
| Day 35 | 54.9 | 51.5 | 54.3 | 52.1 | 3.3 |  |  |  |  |  |  |  |
| Day 45 | 50.3 | 46.3 | 44.9 | 41.5 | 2.3 |  |  |  |  |  |  |  |
| Day 51 | 48.7 | 42.9 | 46.7 | 43.5 | 2.1 |  |  |  |  |  |  |  |

^1^SEM= standard error of the mean.

**Supplementary Table 7.** Effects of source (direct transport from the dairy farms (DF) and subsequent separate housing per dairy farm or transport via a collection center (CC) and subsequent mixed housing) and the prophylactic application of an antibiotic treatment in early-life (AB; yes or no) on immune variables blood of calves obtained via FACS analysis (values are reported as raw means and their pooled SEM^1^).

|  | Dairy farm (DF) | | Collection center (CC) | |  | *P*-value | | | | | |
| --- | --- | --- | --- | --- | --- | --- | --- | --- | --- | --- | --- |
| Parameters | AB | No AB | AB | No AB | SEM | Source | AB | Day | Source×AB | Source×Day | AB×Day |
| CD45cells/ml |  |  |  |  |  | 0.05 | 0.62 | <0.01 | 0.94 | <0.01 | 0.26 |
| Day 1 | 4,532,910 | 4,482,739 | 2,398,914 | 3,210,038 | 569,156 |  |  |  |  |  |  |
| Day 7 | 448,537 | 544,770 | 447,522 | 450,032 | 33,239 |  |  |  |  |  |  |
| Day 21 | 503,112 | 475,420 | 517,707 | 445,137 | 34,167 |  |  |  |  |  |  |
| Day 35 | 504,049 | 432,223 | 533,371 | 481,892 | 51,692 |  |  |  |  |  |  |
| Day 45 | 392,846 | 413,199 | 393,786 | 350,925 | 27,149 |  |  |  |  |  |  |
| CD8cells/ml |  |  |  |  |  | 0.73 | 0.42 | <0.01 | 0.40 | <0.01 | 0.11 |
| Day 1 | 517,439 | 545,041 | 348,543 | 384,647 | 79,506 |  |  |  |  |  |  |
| Day 7 | 47,368 | 65,463 | 60,273 | 61,900 | 5,682 |  |  |  |  |  |  |
| Day 21 | 54,063 | 52,591 | 68,409 | 51,971 | 5,600 |  |  |  |  |  |  |
| Day 35 | 56,215 | 50,567 | 72,464 | 52,526 | 7,510 |  |  |  |  |  |  |
| Day 45 | 44,106 | 49,633 | 49,163 | 37,571 | 3,727 |  |  |  |  |  |  |
| WC1cells/ml |  |  |  |  |  | 0.18 | 0.95 | <0.01 | 0.74 | 0.04 | 0.11 |
| Day 1 | 1,313,731 | 1,314,001 | 672,337 | 834,706 | 215,946 |  |  |  |  |  |  |
| Day 7 | 116,762 | 152,888 | 114,865 | 101,484 | 18,152 |  |  |  |  |  |  |
| Day 21 | 107,335 | 110,906 | 109,338 | 90,356 | 15,173 |  |  |  |  |  |  |
| Day 35 | 85,588 | 79,505 | 89,127 | 61,731 | 13,538 |  |  |  |  |  |  |
| Day 45 | 72,849 | 82,861 | 62,833 | 59,766 | 10,584 |  |  |  |  |  |  |
| CD4 cells/ml |  |  |  |  |  | 0.35 | 0.36 | <0.01 | 0.17 | 0.47 | 0.76 |
| Day 1 | 549,045 | 820,141 | 541,365 | 649,270 | 136,397 |  |  |  |  |  |  |
| Day 7 | 87,031 | 126,205 | 120,767 | 121,515 | 8,237 |  |  |  |  |  |  |
| Day 21 | 88,257 | 114,107 | 120,383 | 109,752 | 10,008 |  |  |  |  |  |  |
| Day 35 | 76,473 | 94,262 | 106,024 | 92,619 | 12,192 |  |  |  |  |  |  |
| Day 45 | 40,124 | 60,388 | 68,726 | 50,327 | 7,088 |  |  |  |  |  |  |
| CD335cells/ml |  |  |  |  |  | 0.25 | 0.53 | <0.01 | 0.18 | 0.01 | 0.47 |
| Day 1 | 195,375 | 183,831 | 95,354 | 146,806 | 32,339 |  |  |  |  |  |  |
| Day 7 | 16,979 | 15,162 | 10,480 | 13,777 | 3,001 |  |  |  |  |  |  |
| Day 21 | 20,042 | 14,213 | 15,401 | 16,752 | 2,310 |  |  |  |  |  |  |
| Day 35 | 15,949 | 11,424 | 13,501 | 15,878 | 1,984 |  |  |  |  |  |  |
| Day 45 | 13,558 | 10,355 | 11,116 | 11,977 | 1,141 |  |  |  |  |  |  |

^1^SEM= standard error of the mean
